# Tumor Arrests DN2 to DN3 Pro T Cell Transition and Promotes Its Conversion to Thymic Dendritic Cells by Reciprocally Regulating Notch1 and Ikaros Signaling

**DOI:** 10.3389/fimmu.2020.00898

**Published:** 2020-06-05

**Authors:** Ipsita Guha, Avishek Bhuniya, Divanshu Shukla, Ashok Patidar, Partha Nandi, Akata Saha, Shayani Dasgupta, Nilanjan Ganguly, Sweta Ghosh, Arathi Nair, Subrata Majumdar, Bhaskar Saha, Walter J. Storkus, Rathindranath Baral, Anamika Bose

**Affiliations:** ^1^Department of Immunoregulation and Immunodiagnostics, Chittaranjan National Cancer Institute (CNCI), Kolkata, India; ^2^Department of Pathogenesis and Cell Responses, National Centre for Cell Sciences, Pune, India; ^3^Department of Molecular Medicine, Bose Institute, Kolkata, India; ^4^Department of Immunology, University of Pittsburgh School of Medicine, Pittsburgh, PA, United States

**Keywords:** thymus, T cell, IL-10, DN2b, DC, Cancer

## Abstract

Tumor progression in the host leads to severe impairment of intrathymic T-cell differentiation/maturation, leading to the paralysis of cellular anti-tumor immunity. Such suppression manifests the erosion of CD4^+^CD8^+^ double-positive (DP) immature thymocytes and a gradual increase in CD4^−^CD8^−^ double negative (DN) early T-cell progenitors. The impact of such changes on the T-cell progenitor pool in the context of cancer remains poorly investigated. Here, we show that tumor progression blocks the transition of Lin^−^Thy1.2^+^CD25^+^CD44^+^c-Kit^low^DN2b to Lin^−^Thy1.2^+^CD25^+^CD44^−^c-Kit^−^DN3 in T-cell maturation, instead leading to DN2-T-cell differentiation into dendritic cells (DC). We observed that thymic IL-10 expression is upregulated, particularly at cortico-medullary junctions (CMJ), under conditions of progressive disease, resulting in the termination of IL-10R^high^ DN2-T-cell maturation due to dysregulated expression of Notch1 and its target, CCR7 (thus restricting these cells to the CMJ). Intrathymic differentiation of T-cell precursors in IL-10^−/−^ mice and *in vitro* fetal thymic organ cultures revealed that IL-10 promotes the interaction between thymic stromal cells and Notch1^low^ DN2-T cells, thus facilitating these DN2-T cells to differentiate toward CD45^+^CD11c^+^MHC-II^+^ thymic DCs as a consequence of activating the Ikaros/IRF8 signaling axis. We conclude that a novel function of thymically-expressed IL-10 in the tumor-bearing host diverts T-cell differentiation toward a DC pathway, thus limiting the protective adaptive immune repertoire.

## Introduction

Immune system decline, dysfunction, and senescence are commonly observed in the setting of cancer progression, with a pronounced restriction amongst CD8^+^ T effector cells, which are known for their capacity to mediate tumor regression (1). T-cell development occurs primarily in the thymus, even in adults, despite the convention for thymic atrophy post-adolescence (2). Interestingly, several recent reports, including a study by Martinez et al., suggest the maintenance of essential thymopoiesis and T-cell neogenesis in adults, which supports the rejuvenation of the peripheral naïve T-cell pool under pro-inflammatory conditions (3, 4). Moreover, adult thymopoiesis has also been reported to increase after growth hormone therapy (4) and pharmacologic androgenic blockade (5). Furthermore, in HIV-infected patients, thymus-derived CD4^+^ T cells are known to increase in frequency after antiretroviral therapy (6). In stark contrast, in tumor-bearing hosts, the thymus may contribute principally to the development of regulatory T cells at the expense of effector T cells (7) and/or to the interruption of CD4^+^CD8^+^ DP immature thymocyte programming (8, 9).

The process of T-cell development starts with the migration of CD4 and CD8 negative—the double negative (DN)-lymphoid progenitor cells—from bone marrow to thymus. During progressive differentiation of DN cells, these cells move from the cortico-medullary junction (CMJ) to the sub-capsular region under the direction of chemokine gradients, where they interact with distinct populations of cortical stromal cells (10). Defined by their differential surface expression of CD25, CD44, and c-Kit, DN cells mature through four stages: DN1 (CD25^−^CD44^+^), DN2 (CD25^+^CD44^+^), DN3 (CD25^+^CD44^−^), and DN4 (CD25^−^CD44^−^). During their migration within the thymic cortex, DN4 cells are converted into CD4^+^CD8^+^ double-positive (DP) thymocytes, which are subsequently positively selected for self-MHC restriction. Finally, after negative selection, mature CD4^+^ or CD8^+^ T cells in medulla [i.e., single-positive (SP) cells], exit the thymus and enter the peripheral circulation (11, 12). This entire T-cell developmental process occurring within the thymus is strictly regulated by thymic cytokines, chemokines, and a coordinated crosstalk between several transcription factors, including Notch1, TCF, Ikaros, and Pu.1, among others (13–19).

Progressive tumor manifests several immune dysfunctions, including thymic atrophy and cessation of effector T-cell functions. Particularly, CD8^+^ T cells in tumor hosts show a broad spectrum of dysfunctional states, shaped by various systemic and intra-tumoral suppressive mechanisms. Among these mechanisms, upregulation of PD1, CTLA4, etc. (20, 21) on the T-cell surface and conversion of T cells to Tregs has emerged as an important contributor, which is reflected in decreased effector T cells in response to tumor antigens, thus causing failure of therapy and tumor progression. Moreover, the elicited insults to T-cell function have been found to be both quantitative and qualitative. In GBM patients, a significant deficiency in the production of mature T cells is observed along with thymic involution (22). Recent studies on lymphoma patients also revealed the existence of T-cell dysfunction, with reduced output of thymic emigrants from atrophied thymus (23, 24) suggesting that tumor cell-secreted factors might contribute to blocking intra-thymic T-cell development. In transplantable T-cell lymphoma, murine hosts show tumor growth-dependent immunosuppression, which is correlated well with a block in early T-cell development in atrophied thymus (25). However, a detailed understanding of the role of tumor burden on early T-cell development and the fate of these targeted pre-T cells is lacking.

Herein, we show that progressive growth of tumors in mice leads to a blockade in the transition from the CD25^+^CD44^+^c-Kit^+^ DN2 stage to the CD25^+^CD44^−^c-Kit^−^ DN3 stage of the T-cell maturation program. The decision-making genes like *notch1* (essential for T-cell lineage commitment) become downregulated, and *ikaros/irf8/pu.1* (essential for DC commitment) become upregulated in DN2a, which instruct the conversion to DC instead of T-cell lineage commitment. This process is driven by increased thymic production of IL-10 under tumor condition, which acts on IL-10R^high^ DN2 cells by promoting DC lineage commitment (with assistance from CD45^−^keratin5^high^ thymic stromal cells). This process differentially regulates *notch1* and *ikaros/irf8* gene transcription in DN2a cells. Tumor-induced IL-10 promotes STAT3 phosphorylation, its subsequent nuclear translocation and binding to *notch1* promoter to silence *notch1* gene transcription. Furthermore, we show that physical contact of IL-10-educated stromal cells with T cells is essential for early T-cell differentiative arrest and the co-option of these precursor cells for differentiation into DC.

## Materials and Methods

### Antibodies and Reagents

RPMI-1640, RF10 (RPMI-1640 + 20 mM HEPES), DMEM high-glucose, and fetal bovine serum (FBS) were purchased from Hi-Media (Mumbai, India). Anti-mouse biotin-conjugated antibodies (lineage cocktail–biotin, and Thy1.2-biotin), anti-mouse fluorescence conjugated antibodies (CD4-FITC, CD8-PE, CD44-FITC, CD25-PE, c-Kit- PE/cy5.5 MHCII-FITC, and CD11c-PE), purified anti-mouse antibodies (CD4, CD8, CD45, Ki67, STAT3, IKAROS, IRF8, IL-10, and IL-10R), and CytoFix/CytoPerm solutions were procured from BD-Pharmingen or Biolegend (San Diego, CA, USA). Anti-pSTAT3 antibody and rmIL-10 were purchased from BD Biosciences (San Jose, CA). Aminoethylcarbazole (AEC) chromogen solution, and aqueous mounting media were procured from VECTOR Laboratories Inc. (Burlingame, CA).

### Mice and Tumor

Wild-type (Wt) female C57BL/6 and Swiss mice (age: 4–6 weeks, body weight: 20–25 g on average) were obtained from Animal Facilities of the National Institute of Nutrition (Hyderabad, India). IL-10^−/−^ mice were procured from Jackson Laboratories (Bar Harber, ME) and subsequently bred at the National Center for Cell Science (Pune, India). The care of animals was carried out according to the guidelines established by the Institutional Animal Care and Ethics Committee (IAEC Approval No. IAEC-1774/RB-4/2015/6 and IAEC-1774/RB-19/2017/15). Autoclaved dry pellets and water were provided *ad libitum*.

### Tumor Growth and Development

Tumor-bearing mice were euthanized by overdose of Ketamine HCl (160 mg/kg) + Xylazine (20 mg/Kg) by intraperitoneal injection. They were euthanized if tumor size reached 20 mm in either direction, if the animal looked sick, or if any necrosis of tumor was observed. The overall health of animals was monitored twice a day and once in holidays. Animal death and abnormal symptoms, if any, were recorded thoroughly. Mice were monitored and cared for according to the guidelines established by the Institutional Animal Care and Ethics committee, CNCI, Kolkata.

### Tumor Growth Measurement

Swiss and C57BL/6 mice (*n* = 10 in each group; two groups; normal and tumor) were inoculated s.c. with syngenic sarcoma 180 (1 × 10^6^ cells/mice), B16F10 melanoma (2 × 10^5^ cells/mice), or Lewis lung carcinoma (LLC) (2 × 10^5^ cells/mice) cells in the lower right flank to establish solid tumors. Tumor growth was then monitored bi-weekly using calipers. Tumor size was recorded in mm^2^ (as the product of length × width), and mice were sacrificed after euthanasia when tumor had reached a size of 20 mm in any direction.

### Fetal Thymic Organ Culture (FTOC)

Mice fetal thymic pieces (Gestation period E14.5) were dissected and cultured as previously described for a total of 7 days [(26), Supplementary Figure 4]. Dissected thymic lobes were placed on 0.8 μm membrane on an anti-wrap sponge at the liquid/air interface in six-well plates containing 2 ml of medium (DMEM high-glucose supplemented with 10% FBS, glutamine, and penicillin/streptomycin). Twenty-four hours later, GSI 953 (CPD11, 50 μM final concentration, delivered in DMSO) or the solvent DMSO alone was added at 12-h intervals over the next 3 days (27). After 3 days in culture, 10 ng/ml of rmIL-10 (BD Biosciences, San Diego, USA) was added to the FTOC medium to simulate tumor-induced thymic alterations. Three days later, single cells were prepared by collagenase (1 mg/ml) treatment for phenotypic analysis.

### DN Thymocytes and Thymic Stromal Cell Co-culture

CD4^−^CD8^−^ DN T cells were isolated (>95% pure) from mouse thymus by negative selection using BD IMag Anti-Mouse CD4 and CD8 Particles-DM (BD Biosciences, San Diego, CA). DN-T cells were cultured in complete RPMI-1640 medium (Invitrogen, Camarillo, CA). Fetal thymic organs (FTOs) from wild-type and IL-10^−/−^ were cultured in DMEM high-glucose with 2′-deoxyguanosine (dGuo, Sigma Aldrich, St. Louis, USA) to a final concentration of 1.35 mM for 5 days. After 5 days, fetal thymic lobes were treated with collagenase (1 mg/ml) for single-cell preparation to isolate stromal cells. DN-T cells and stromal cells (28) were pretreated with mrIL-10 (10 ng/ml) for 24 h before their subsequent co-culture for an additional 24 h.

### RT-PCR and Quantitative Real-Time PCR

Cellular RNA was isolated using Trizol (Invitrogen, Camarillo, CA), and random hexamers were used to generate corresponding cDNA (First Strand cDNA Synthesis Kit; Fermentas, Hanover, MD). RT-PCR amplification was performed using 2X Go Taq Green Mix (Promega, Madison, USA), and quantitative real-time PCR was performed by SYBR green (Roche, Germany). PCR was done with the following program: 94°C for 5 min; 35 cycles of 94°C for 30 s, 54–57°C for 30 s, and 72°C for 1 min; 72°C for 5 min. PCR products were identified by image analysis software for gel documentation (Versadoc; BioRad Laboratories, Hercules, CA) after electrophoresis on 1.5% agarose gels and stained with ethidium bromide (Sigma-Aldrich, St Louis, USA). RT-PCR primers were designed and purchased from MWG-Biotech (Bangalore, India). In quantitative PCR, after calculating the Ct value, expression fold change was analyzed.

### Flow-Cytometric Staining

Thymocytes were isolated from normal and tumor-bearing hosts. The lineage negative Thy1.2 positive population was obtained by removal of mature lineage positive cells using cocktails of biotinylated lineage antibodies: anti-B220, anti-TER119, anti-CD11b (Mac-1), anti-Gr-1, and anti-CD3ε (Biolegend, San Diego, CA), followed by negative selection using BD IMag streptavidin particles-DM (BD Biosciences, San Diego, CA); Thy1.2 positive cells were isolated by positive selection using BD IMag anti-mouse Thy1.2 (CD 90.2) biotin streptavidin particles-DM (Biolegend, San Diego, CA), then those cells were sorted based on CD44 and CD25 expression using a FACSAria cell sorter (Becton Dickinson, Mountainview, CA). Flow cytometry was used to determine cell-surface phenotypes after first staining cells (1 × 10^6^) with fluorescently labeled antibodies (specific and isotype-matched controls). After incubation for 30 min at 4°C in the dark, labeled cells were washed twice with FACS buffer (0.1% BSA in PBS) before flow-cytometric analysis. Similarly, intracellular molecules (i.e., Notch1, Ikaros, and IRF8) were stained with anti-mouse fluorescence-labeled antibodies using Cytofix/Cytoperm reagents per the manufacturer's protocol (BD Biosciences, San Diego, CA). For Ki67 staining, 70–80% chilled ethanol was added to fix the pelleted cells (1.5 × 10^7^ cells) with vortexing, followed by incubation at −20°C for 2 h. Fixed cells were then washed twice with staining buffer and centrifuged (10 min, 200 × g), then diluted to a concentration of 1 × 10^7^ cells/ml for staining and corollary flow-cytometry analyses. Cells were then fixed with 1% paraformaldehyde in PBS; acquisition was performed using a FACS Calibur (Becton Dickinson, Mountainview, CA) along with suitable negative isotype controls. For assessment of cellular apoptosis, fixed cells were stained with AnnexinV and PI by FITC-AnnexinV apoptosis detection kit I (BD, Biosciences, San Jose, CA). The percentage of positively stained populations was determined using quadrant statistics established using Cell Quest (Becton Dickinson, Mountainview, CA) and FlowJo software (Tree Star, Ashland, OR).

### Immunohistochemistry of Thymus Section

Thymus tissue samples were prepared (paraffin-embedded and frozen sample), and 5-μm sections were stained, as previously reported (29), with anti-mouse IL-10 antibody (Biolegend, San Diego, USA). Imaging was done by ZEISS Primo Star microscope, Zeiss (Zena, Germany), and laser capture micro-dissection was done by ZIESS PALM/APOSTOME (Zena, Germany) laser capture microscope.

### Fluorescence Imaging of Thymus Sections

Tumor tissue samples were prepared from frozen sections by cryostat sectioning, and 5-μm sections were stained as previously reported (30). FITC-conjugated anti-mouse CD44 and PE-conjugated anti-mouse CD25 or matching isotype controls (all from BD Biosciences, San Jose, CA) were used. Imaging was performed using a ZEISS LSM-710 confocal microscope (Zena, Germany). Images were analyzed by ImageJ software, https://imagej.net>Fiji. The co-localization index was expressed by Mander's coefficient. A value close to 1 indicates reliable co-localization.

### BrdU Labeling-Based Proliferation Assay

For detection of the thymic T-cell proliferation in the presence of tumor conditioning, a BrdU Labeling and Detection Kit I (Roche Diagnostics, Mannheim, Germany) was used per the manufacturer's instructions. Mice were injected intravenously with the BrdU labeling reagent (concentration 10 μM, dose 300 μl/25 gm body weight; two doses with a 4-h interval), with animals euthanized 1 h after the final injection and organs harvested for further studies. BrdU was detected with primary monoclonal anti-BrdU and secondary anti-mouse Ig FITC antibodies. T-cell proliferation was analyzed by flow cytometry.

### siRNA-Mediated STAT3 and Ikaros Silencing

STAT3 siRNA (Santacruz Biotechnology, Dallas, TX) was procured, and Ikaros siRNA was prepared with a Silencer® siRNA construction kit (Life Technologies, USA). For Ikaros siRNA preparation, first sense 5′AATGGGGAAGAATGTGCAGAGCCTGTCTC-3′ and antisense 5′-CTCTGCACATTTCTTCCCCATTCCTGTCTC-3′ primer were taken, and siRNA was prepared as per manufacturer protocol. Both the siRNAs were added in FTOC to a final concentration of 100 nM (50 μM/25 μl). In different experimental setups, siRNA and lipofectamine-2000 reagent (Invitrogen, USA) (6 μl) were added to two Opti-MEM aliquots (250 μl each) and incubated for 5 min at RT (31). The siRNA/Opti-MEM and the Lipofectamine/Opti-MEM (500 μl total volume) were mixed and allowed to incubate for 20 min at RT. siRNA-containing medium was then added to the FTO culture. IL-10 (10 ng/ml) was added to the FTOC medium to mimic the tumor-induced thymic alteration. Finally, STAT3 and Ikaros expression were checked both in untreated and siRNA-transfected FTOCs by FACS staining. In siRNA-treated cells, STAT3 expression was confirmed to be reduced to 30% and Ikaros expression to be reduced to 50% of the control siRNA-treated cells.

### CD49d-Mediated Inhibition of Extra-Thymic DC Homing

CD49d (Integrin-4α) neutralizing antibody (Invitrogen, California, USA) was used to inhibit extra-thymic DC homing in tumor host. Tumor-bearing mice were injected intra-peritoneally with the CD49d neutralizing antibody (concentration 1 mg/ml, dose 62.5 μg/25 gm body weight; 4 doses with 48 h interval over a period of 8 days), with animals euthanized the day after the final injection and organs harvested for further studies.

### Statistical Analysis

All reported results represent the mean ± SE of data obtained in either 4–6 (for *in vivo* analysis) or 3–6 (*in vitro* assays) independent experiments. Statistical significance was determined using an unpaired *t*-test and one-way ANOVA followed by Tukey's multiple comparison in INSTAT3 Software (Graphpad, CA, USA). Differences between groups attaining a *p* < 0.05 are considered significant.

## Results

### Tumor-Induced Thymic Atrophy Is Associated With the Early Arrest of T-Cell Differentiation

**Figure 1:**
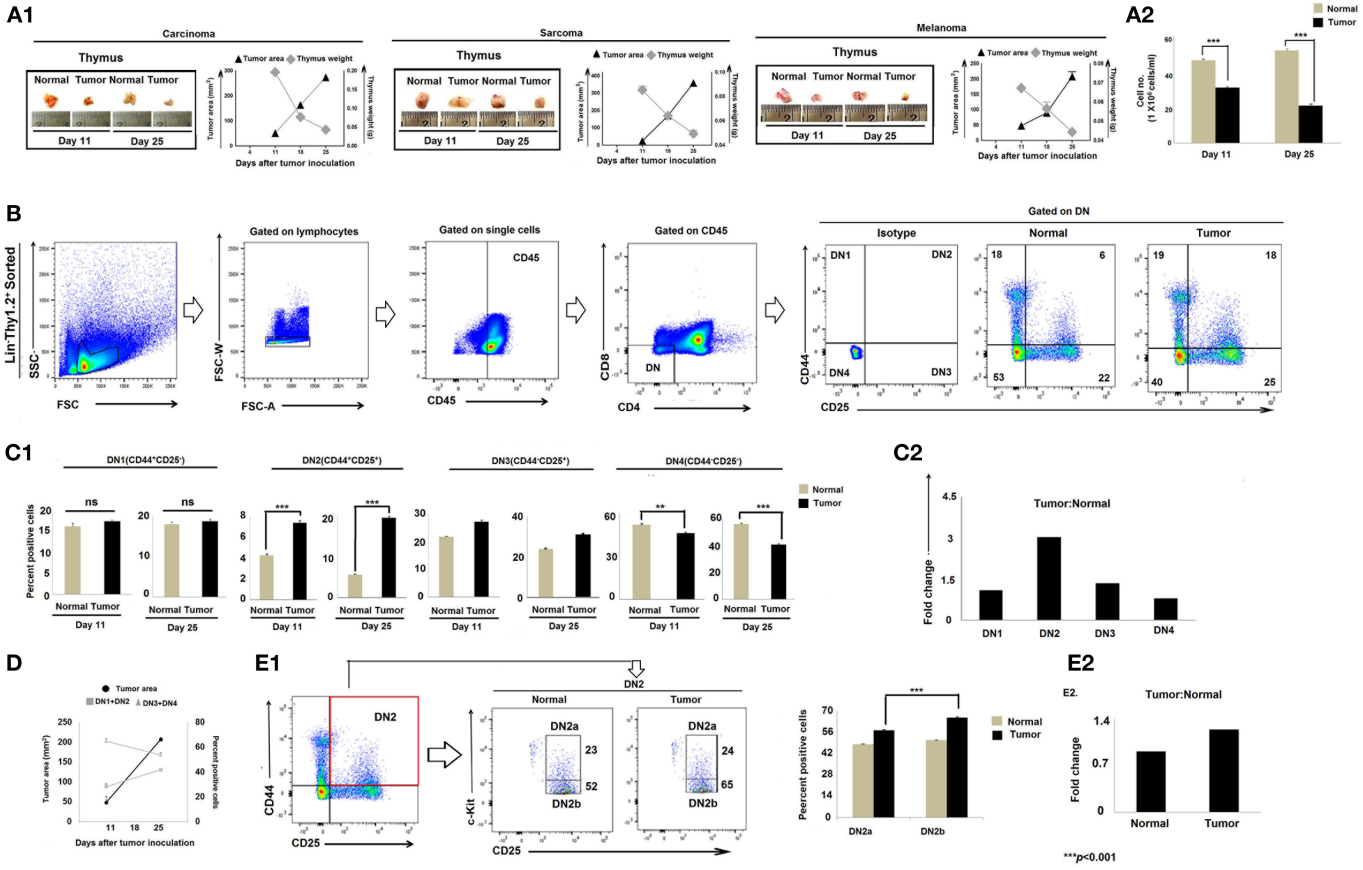
Tumor-induced thymic atrophy is associated with arrest of DN pro-T cell maturation. **(A1)** Representative figures of thymi normal and tumor (carcinoma, melanoma, and sarcoma)-bearing mice on days 11 and 25 after tumor inoculation, along with line diagrams of tumor area (mm^2^) vs. thymic weight (gm); *n* = 12 in each case. **(A2)** Bar diagram representing total thymocyte number from normal and tumor-bearing mice; *n* = 12 in each case. **(B)** Thymic T cells sorted based on Lin^−^ Thy1.2^+^ phenotype and flow-cytometric analysis of CD25 and CD44 represent DN1 to DN4 T cells of normal and tumor-bearing mice. **(C1)** Bar diagrammatic representations of mean ± SE of positive percentage of Lin^−^Thy1.2^+^ DN1 (CD25^−^CD44^+^), DN2 (CD25^+^CD44^+^), DN3 (CD25^+^CD44^−^), and DN4 (CD25^−^CD44^−^) T cells, respectively, from normal and tumor-bearing mice; *n* = 4 in each case, ***p < 0.001. **(C2)** Bar diagrammatic representation of the fold change of the DN1 to DN4 population of tumor:normal, *n* = 4. **(D)** Line diagram of percentage positive T cells (DN1+DN2 vs. DN3+DN4) and tumor area (mm^2^) of tumor-bearing mice on different days after tumor inoculation. **(E1)** Flow-cytometric representations of Lin^−^Thy1.2^+^-sorted T cells with CD25, CD44, and c-Kit staining, indicating the percentage of DN2a and DN2b cells. Bar diagrammatic representation of positive percentage of DN2a (CD25^+^CD44^+^c-Kit^high^) and DN2b (CD25^+^CD44^+^c-Kit^low^) cells from normal and tumor hosts; mean ± SE; *n* = 4 in each case, ***p < 0.01. **(E2)** Bar diagrammatic representation of the fold change of DN2a:DN2b expression from normal and tumor-bearing mice, *n* = 4.

Immune suppression in the cancer-bearing host has previously been associated with involution or atrophy of the thymus, the primary site of T-cell development and education (32, 33). We confirmed thymic atrophy in the face of tumor progression in three mouse tumor models, including lung carcinoma (Lewis lung), sarcoma (S180), and melanoma (B16F10) (Figures 1A1, A2). Because T-cell development begins with CD4^−^CD8^−^DN pro-T cells that subsequently pass through four well-defined maturation stages, we next analyzed Lin^−^Thy1.2^+^DN subpopulations based on their differential expression of CD25 (IL-2Rβ), CD44 (pg1), and CD117 (c-Kit). Flow-cytometric analyses revealed significant alterations in the Lin-Thy1.2^+^ DN2, DN3, and DN4 subpopulations (CD25^−^CD44^+^c-Kit^−^ DN1, CD25^+^CD44^+^c-Kit^+^ DN2, CD25^+^CD44^−^c-Kit^−^ DN3, and CD25^−^CD44^−^c-Kit^−^ DN4) isolated from the thymi of tumor-bearing mice vs. control tumor-free mice (Figures 1B,C). As shown in Figures 1B–D, the proportions of early T-cell progenitors (DN1 to DN3 and particularly DN2) were markedly increased in tumor-bearing mice, while late-stage T-cell progenitors (DN4) were decreased, implying a blockade in T-cell precursor transition through normal differentiation programming. These alterations became more pronounced on day 25 of tumor growth (mean tumor size, 250–270 mm^2^) when compared to day-11 tumors (mean tumor size, 70–80 mm^2^) (Figures 1C,D). In order to identify the exact stage of the blockade in DN-T-cell transition, we next examined the status of the DN2a (CD25^+^CD44^+^c-Kit^high^) and DN2b (CD25^+^CD44^+^c-Kit^low^) subpopulations within the tumor-conditioned thymi. We observed that, in tumor-bearing mice, the DN2-to-DN3 transition was arrested (Figures 1E1,E2). These results suggest that progressive tumor growth restricts the early stages of T-cell differentiation in the thymus at the DN2b-to-DN3 transition stage.

### Accumulation of Early DN2 Pro-T Cells in Tumor-Bearing Mice Is Not Associated With Enhanced Proliferation of DN2 Cells or Enhanced Apoptosis of DN3 Cells

**Figure 2:**
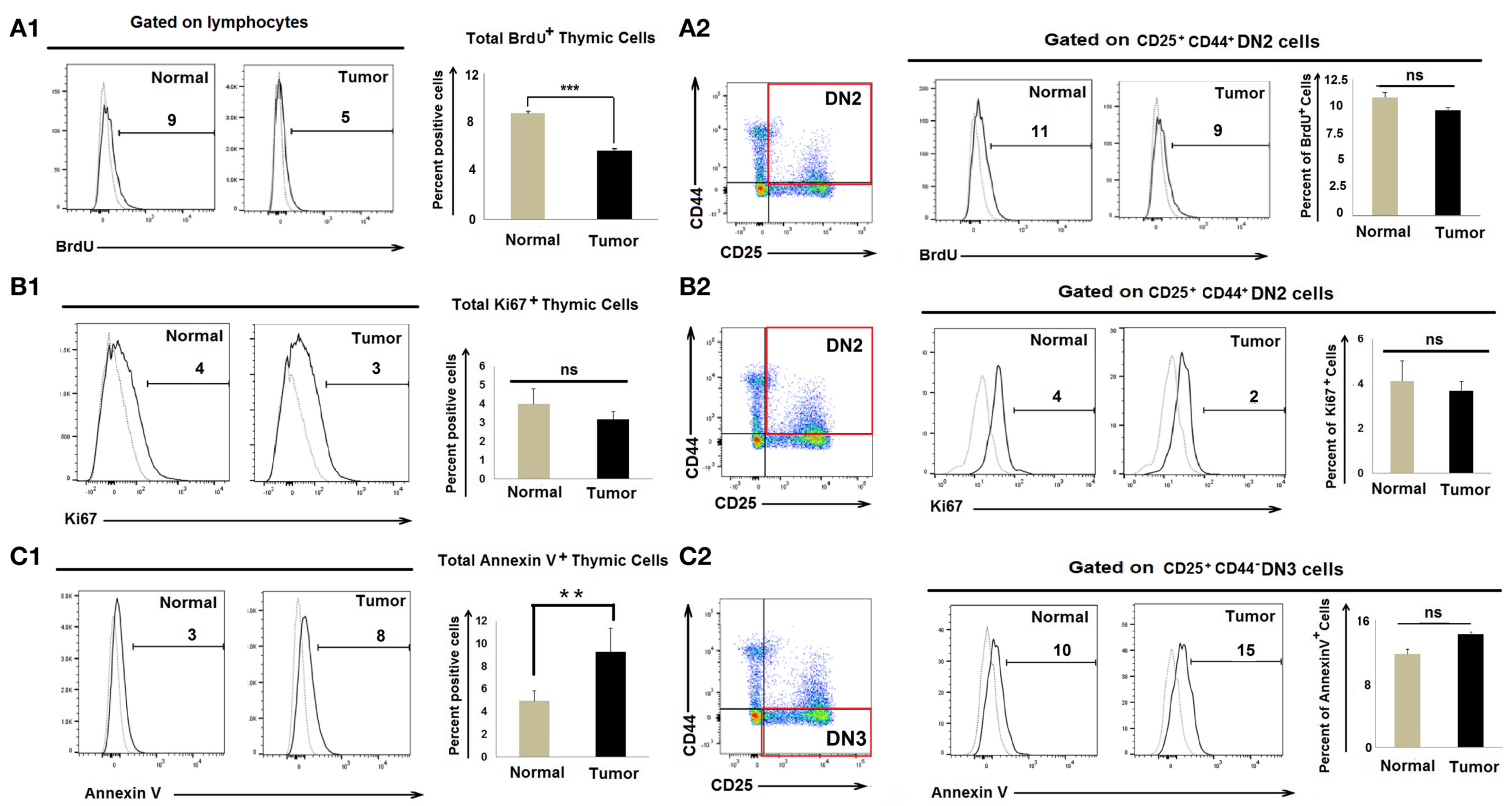
Accumulation of DN2b T cells associated with neither proliferation nor apoptosis: Flow cytometric analysis as presented by histograms and bar diagrams of total BrdU **(A1)**, Ki67^+^ **(B1)**, and AnnexinV^+^ **(C1)** cells from thymi of normal and tumor-bearing mice. Dotted line represents isotype control. Similar analysis of CD25^+^CD44^+^BrdU^+^ **(A2)**, CD25^+^CD44^+^Ki67^+^ **(B2)**, and CD25^+^CD44^+^AnnexinV^+^ **(C2)** cells from normal and tumor-bearing mice, where dotted line represents FMO; mean ± SE are shown in bar diagrams (*n* = 3 in each case); **p < 0.01, ***p < 0.001.

As numbers of CD25^+^CD44^+^c-kit^+^ DN2 cells increase in tumor-bearing mice, we evaluated whether tumor progression led to enhanced DN2-cell proliferation. Control and tumor-bearing mice were injected twice with BrdU (300 μl/25 gm body weight at 4-h intervals), and thymi were harvested 1 h after the final injection. CD25^+^CD44^+^ DN2 cells stained with an anti-BrdU detection antibody indicated that there was a modest decrease in total BrdU^+^ thymic cells in tumor progressors; however, proliferation amongst BrdU^+^ DN2 cells remained unchanged in control vs. tumor-bearing mice (Figure 2A). As a confirmatory analysis, expression of the nuclear proliferation antigen Ki67 was used as an endpoint index in flow cytometry assay, which also revealed comparable DN2-cell proliferation in control vs. tumor-bearing mice (Figure 2B).

Since numbers of DN3 cells waned in tumor-bearing mice, we next examined whether the reduction in DN3 cells in tumor-conditioned mice was due to enhanced rates of apoptosis. We observed insignificant changes in Annexin-V^+^ DN3 populations in the tumor-bearing mice when compared to tumor-free control mice (Figure 2C). These observations ruled out the possibility that heightened proliferation or enhanced apoptosis accounts for the observed increase in DN2 cells or the reduced count of DN3 cells, respectively, in the thymus of tumor-bearing mice.

### CCR7 Downregulation Is Associated With Impaired DN2-to-DN3 Transition in Tumor-Bearing Mice

**Figure 3:**
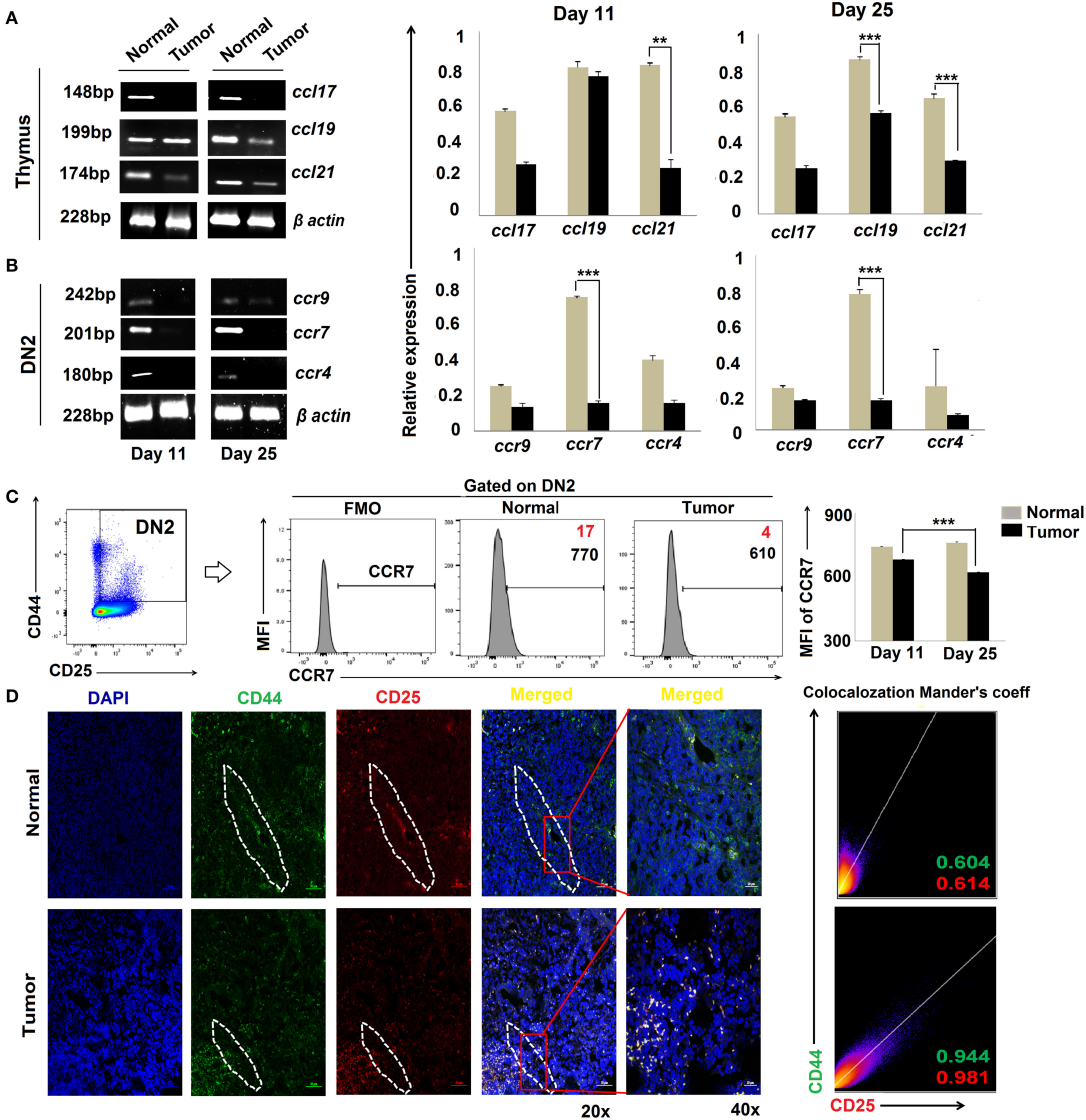
Altered chemokine and chemokine ligand expressions are associated with tumor induced-arrest of DN2-to-DN3 transition: **(A)** Total thymic cells from normal and tumor hosts on days 11 and 25 (*n* = 4 in each group) were isolated to purify mRNA, and expressions of different chemokine ligands (*ccl17, ccl19*, and *ccl21)* were assessed by RT-PCR, keeping β-actin as a loading control. Bar diagrams show the mean ± SE of expression of *ccl17, ccl19*, and *ccl21* from normal and tumor host on days 11 and 25, respectively (*n* = 3); **p < 0.01, ***p < 0.001. **(B)** Total thymic sorted DN2 (CD25^+^CD44^+^) cells were used to isolate mRNAs and analyzed by RT-PCR for different chemokine genes, e.g., *ccr9, ccr7*, and *ccr4*, from normal and tumor hosts on days 11 and 25 (*n* = 4 in each group), keeping β-actin as a loading control. Representative gene expression pattern is shown in panels. Bar diagrams show the mean ± SE of expression of *ccr9, ccr7*, and *ccr4* from normal and tumor host on days 11 and 25 (*n* = 4); **p < 0.01, ***p < 0.001. **(C)** Flow-cytometric analysis of CD25^+^CD44^+^CCR7^+^ thymic cells from normal and tumor-bearing mice. Representative histograms for CCR7 on CD25^+^CD44^+^(DN2) gated cells, and bar diagrammatic representation shows the mean ± SE of percentage positive CD25^+^CD44^+^CCR7^+^ cells, where positive percentages of cells are shown in red, and bar diagram represents the mean ± SE of MFI of CD25^+^CD44^+^CCR7^+^ cells; *n* = 4 in each group; ***p < 0.001. **(D)** Immunofluorescence staining was performed with CD25-PE and CD44-FITC on thymuses from normal and tumor hosts. Representative DAPI, CD44-FITC, CD25-PE, and merged figures of stained tissues show single staining and the co-localization of CD25 and CD44 in tissues from normal and tumor hosts. Dotted regions indicate the CD44^+^CD25^+^-localized CMJ regions in both cohorts. Representative 2D intensity histogram represents co-localization of CD25 and CD44; Manders' co-efficient represents the intensity and index of co-localization. Value close to 1 indicates reliable co-localization.

Migration of T-cell progenitors through a distinct stromal microenvironment is required for sequential interactions with cortical and medullary stromal cells that educate T cells during their development in the thymus (34). Given our findings for DN2 → DN3 arrest in tumor-bearing mice, we next evaluated the expression of chemokines and their corresponding receptors associated with the trafficking of pro-T cells within the thymic microenvironment. Expressions of various CC, CXC chemokines and respective receptors like CCR4, CCR9, and CCR7, etc., were studied using RT-PCR or flow cytometry. Analysis of the total thymic cell population suggests insignificant changes in CCL17 (ligand for CCR4) and a modest decrease in CCL19 (ligands for CCR7) at day 25 but a drastic reduction in expression of CCL21 (ligands for CCR7) when comparing thymi harvested from tumor-bearing vs. control mice (Figure 3A). Expression of chemokine receptors also varied greatly among the different subpopulations of DN cells isolated from the thymus of tumor-bearing vs. control animals. Notably, the expression of CCR7 was strongly reduced in association with tumor progression in DN2 cells at both the transcript and protein levels (Figures 3B,C) while remaining unchanged in the DN1 and DN4 subsets (*data not shown*). Under normal conditions, *ccr7* expression was highest in the DN2 and DN3 subsets and lowest in the DN1 and DN4 subsets. Remarkably, immunofluorescence analyses revealed a more pronounced localization of CD25^+^CD44^+^ DN2 T cells at cortico-medullary junctions of thymus in tumor-bearing vs. tumor-free mice (Figure 3D). These data suggest significant alterations in CCR7 expression at the DN2 stage and expression of CCR7 ligands (CCL19 and CCL21) in the thymus that may be associated with arrest in T-cell maturation in the tumor-bearing host.

### DN2b Cells Are Diverted to DC Programming Based on Suppressed Expression of Notch1 and Increased Expression of Ikaros in DN2a Cells

**Figure 4:**
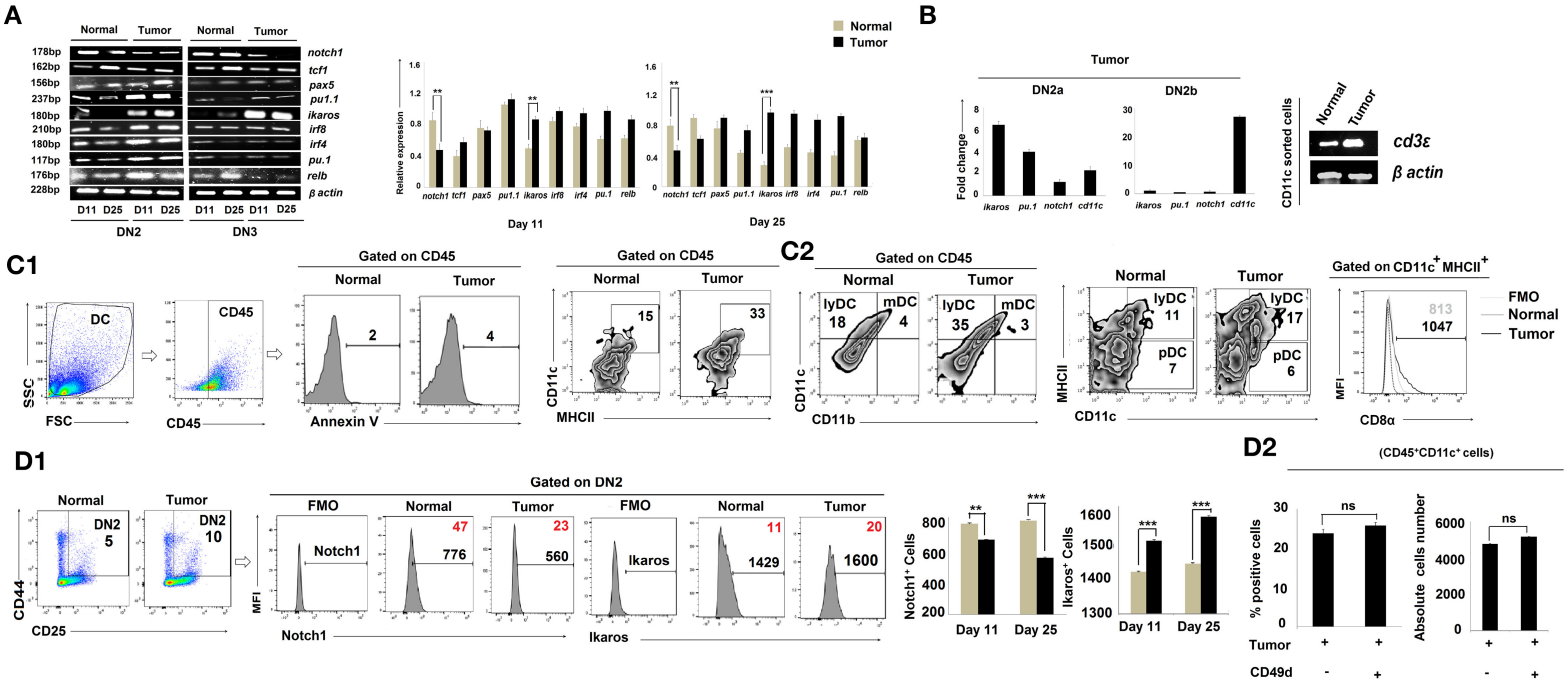
Tumor microenvironment induces DC lineage commitment of DN2 pro-T cells. **(A)** Thymic T cells from normal and tumor hosts were sorted for CD25^+^CD44^+^ and CD25^+^CD44^−^ cells as DN2 and DN3 by flow cytometry on days 11 and 25 after tumor inoculation. Different gene expressions for different transcription factors were checked by RT-PCR using mRNA from thymic T cells, keeping β-actin as a loading control. *notch1* and *tcf1* were checked for T cells, *pax5* for B cells, *pu1.1* for macrophages, and *ikaros, irf8, irf4, pu.1*, and *relb* for myeloid lineage commitment. Representative expression patterns of genes are presented in the left panel along with bar diagrams showing mean ± SE of expression of mentioned transcription factors from normal and tumor hosts on days 11 and 25, respectively (*n* = 4); **p < 0.01, ***p < 0.001. **(B)** Thymic T cells from normal and tumor hosts were sorted for CD25^+^CD44^+^c-Kit^high^ and CD25^+^CD44^+^c-Kit^low^ cells as DN2a and DN2b by flow-cytometry on day 25 after tumor inoculation. From the mRNA, different gene expressions were checked by quantitative real-time PCR, keeping β-actin as a housekeeping gene. Ct values were calculated, and fold change of Ct value against experimental control was represented by mean ± SE in a bar diagram (*n* = 3). The right panel shows the gene expression pattern of *cd3*ε from CD11c^+^-sorted thymic cells from normal and tumor hosts using β-actin as a loading control. **(C1,C2)** The total percentages of dendritic cells of normal and tumor-bearing mice were checked by flow cytometry. Thymic cells were stained with CD45, Annexin V, CD11c, MHCII, CD11b, and CD8α. Gating strategies of staining are shown, and flow-cytometric representations of CD45^+^CD11c^+^MHCII^+^, CD45^+^CD11b^−^CD11c^+^ (lymphoid DC), CD45^+^CD11b^+^CD11c^+^ (myeloid DC), CD11c^+^MHCII^high^CD8α^+^ (lymphoid DC), and CD11c^+^MHCII^low^CD8α^+^ (Plasmocytoid DC) cells reveal the number of total dendritic cells. **(D1)** Further validation of Notch1 and Ikaros expression was performed by flow cytometry along with DN2 (CD25, c-Kit) markers from thymic T cells. In histograms, positive percentages of cells are in red. Bar diagrams represent mean ± SE of percentage of DN2^+^Notch1^+^and DN2^+^Ikaros^+^ (*n* = 4) cells, respectively; **p < 0.01, ***p < 0.001. **(D2)** Increased population of thymus from tumor host was checked after CD49d treatment (4 doses with 48 h interval over a period of 8 days). CD45^+^CD11c^+^ dendritic-cell population of thymus from B16 tumor host with or without CD49d treatment was checked by flow cytometry. Bar diagrams represent mean ± SE of percentage positive CD45^+^CD11c^+^ dendritic-cell population (left panel) and absolute cell numbers (right panel) of CD45^+^CD11c^+^ thymic cells from tumor host.

CCR7 expression is regulated by Notch1, a known controller of thymic pro-T-cell development (35, 36). Given our finding of reduced expression of *ccr7* in the tumor-conditioned DN2 subpopulation and the arrest of these cells for further T-cell lineage commitment and maturation, we next examined *notch1* expression amongst the various DN subpopulations. RT-PCR analysis of sorted DN2 cells revealed a significant loss of *notch1* gene expression in thymus on days 11 and 25 in tumor-bearing mice vs. control mice (Figure 4A). These DN2 cells also expressed significantly higher levels of *ikaros, irf8*, and Pu.1 (Figure 4A). Given the increased expressions of *ikaros, irf8*, and *pu*.1 along with decreased expression of *notch1* in the thymic DN2 population of the tumor host, we further analyzed these genes within the flow-sorted-DN2a and DN2b population by quantitative real-time PCR. Expressions of *ikaros* and *pu.1* were significantly elevated and that of *notch1* was decreased in the DN2a population isolated from tumor host compared to the same from normal host, while expression of *cd11c* became upregulated in the DN2b population isolated from tumor host (Figure 4B). Since pu.1 serves a critical role in intrathymic DC development, and both Ikaros and IRF8 are critically associated with extra-thymic lineage commitment of DC, we next surveyed for the composition of lymphoid, myeloid, and plasmacytoid DC subsets within thymus. Flow-cytometric analysis suggested that tumor progression (from days 11 to 25) results in increased frequencies of CD45^+^CD11c^high^CD11b^−^MHCII^high^ lymphoid-DC, with the majority of DCs exhibiting a CD8α^+^ phenotype and expressed a significant amount of *cd3*ε (Figures 4B,C1,C2). Likewise, flow-cytometric analysis also suggested an increased percentage of CD4^−^CD8^−^CD25^+^CD44^+^Ikaros^+^ cells and a downregulation in CD4^−^CD8^−^CD25^+^CD44^+^Notch1^+^ cells within the thymus of tumor-bearing vs. control mice (Figure 4D1). We also checked for expression of other crucial transcription factors required for B-cell, macrophage, and T-cell lineage commitment in the various DN subpopulations. We observed no significant alterations in expression of *pax5* (B cells) and *pu1.1* (macrophages) in the tumor-bearing vs. control cohorts (Figure 4A). Furthermore, there were no significant alterations in the percentages of B cells and macrophages within the thymus, regardless of tumor status in the animals (*data not shown*). However, as shown in Figure 4A, expression of the T-cell lineage commitment marker *tcf1* was decreased in DN2 and DN3 cells sorted from the thymus of day-25 tumor-bearing mice compared to normal control mice. To check the possible contribution of extra-thymic/circulating DC in the enhanced DC pool in the thymus of tumor host, we next treated tumor-bearing mice with a neutralizing antibody for CD49d (37) for 8 days (4 times over a period of 8 days). Although such treatment increases the thymus volume, it failed to affect the number of CD11c^+^ cells within the CD45 gated population (Figure 4D2), thereby excluding the possibility of homing of extrathymic DC. These aggregate data suggest that the reciprocal regulation of Notch1 and Ikaros in DN2 subpopulations in the thymus of tumor-bearing mice instigates early arrest of T-cell development at the DN2a stage and its diversion toward the DC lineage.

### Reciprocal Notch1 and Ikaros Regulation by IL-10 Impacts DN2b T-Cell Arrest

**Figure 5:**
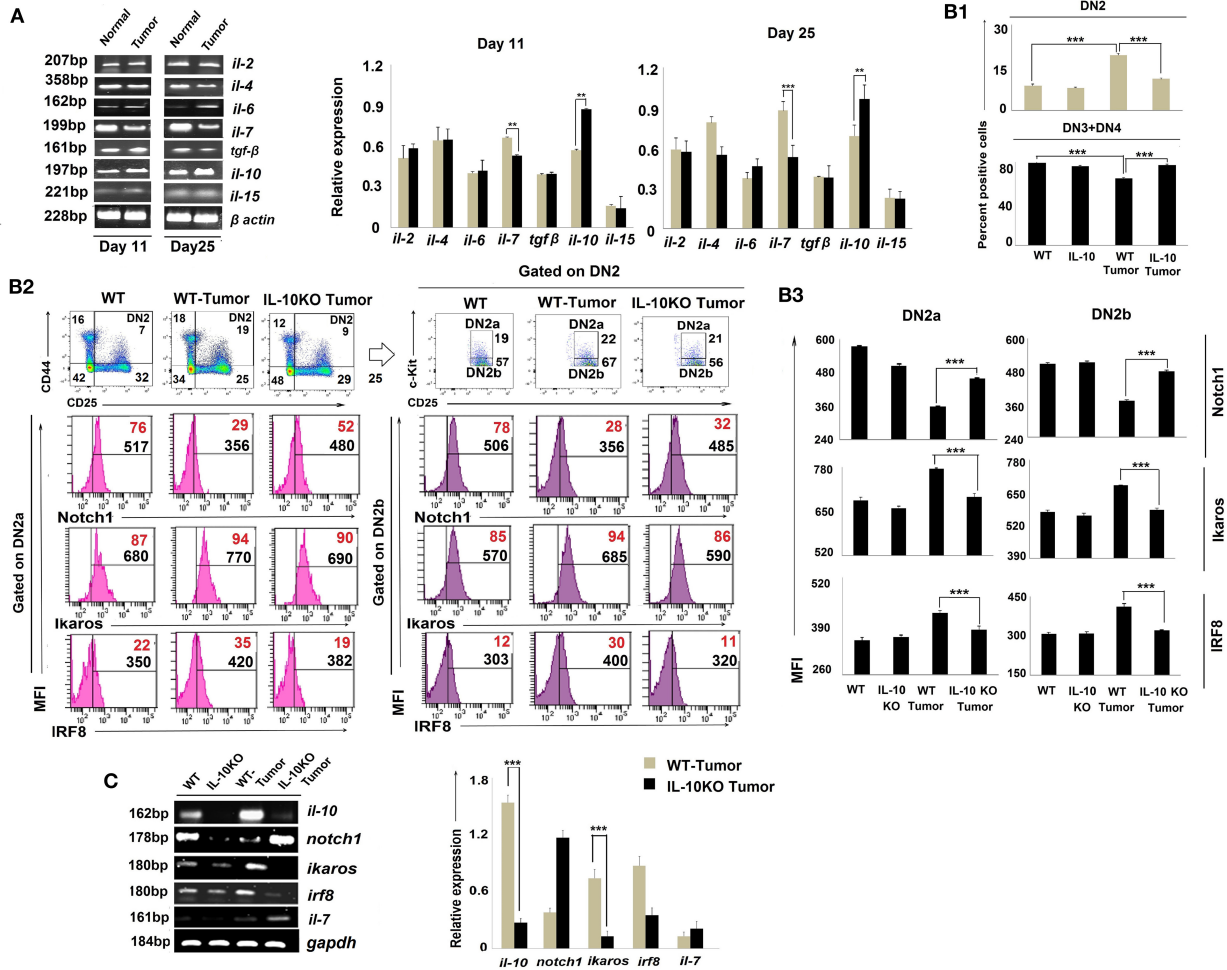
IL-10 promotes tumor-induced early arrest of DN2 pro-T cells by reciprocal regulation of Notch1 and Ikaros signaling. **(A)** mRNA was isolated from thymi of normal and tumor hosts, and cytokine (*il-2, il-4, il-6, il-7, il-10, il-15*, and *tgf*β) profiles were assessed by RT-PCR, keeping β-actin as a loading control. Representative figures of gene expression profile are shown in the left panel and bar diagrammatic representations of mean ± SE of relative expression of above-mentioned cytokines from normal and tumor hosts on days 11 and 25 following tumor inoculation, respectively, are shown in the right panel, *n* = 3; **p < 0.01, ***p < 0.001. **(B1)** Thymic T cells were isolated from non-tumor (wild-type and IL-10^−/−^) and tumor (wild-type and IL-10^−/−^) mice. DN2, DN3, and DN4 cells were stained with CD45, CD4, CD8, CD25, CD44, and c-Kit. Gating strategies are shown in Supplementary Figure 1. Bar diagrams represent the mean ± SE percentage of DN2 and DN3+DN4 positive cells from the above-mentioned mice, respectively. **(B2)** Representative figures of flow cytometric analysis performed with CD45, CD4, CD8, CD44, CD25, c-Kit, Ikaros, IRF8, and Notch1 in thymic T cells from wild-type and IL-10^−/−^ tumor-bearing mice. Histograms show MFI and positive percentages of cells of DN2a^+^Notch1^+^, DN2a^+^ Ikaros^+^, DN2a^+^IRF8^+^ DN2b^+^Notch1^+^, DN2b^+^ ${\text{Ikaros}}_{,}^{+}$ and DN2b^+^IRF8^+^. **(B3)** Bar diagrams showing mean ± SE of MFI values of Notch1^+^, Ikaros^+^, and IRF8^+^ within DN2a and DN2b cells, respectively, from the mice mentioned in B2. **(C)** Cytokine and transcription factor profiles were assessed in sorted DN2 cells from non-tumor (wild-type and IL-10^−/−^) and tumor (wild-type and IL-10^−/−^) mice by analyzing *il-10, notch1, ikaros, irf8*, and *il-7* gene expressions by RT-PCR, keeping gapdh as a loading control. Representative figures along with bar diagram show mean ± SE of different gene expressions from the above-mentioned mice. *n* = 4 in each group; *p*-values are mentioned in figure.

As the cytokine microenvironment within the thymus critically regulates T-cell development (38) and is perturbed by tumor conditioning (39), we assessed cytokines for their role(s) in reciprocally regulating Notch1 and Ikaros/IRF8 expression in arrested T cells. Cytokines relevant to thymic regulation such as *il-2, il-4, il-6, il-7, il-10, il-15*, and *tgf*β were monitored amongst total thymic cell populations using RT-PCR. Our results suggest that tumor-induced arrest of DN2 T cells in the thymus is correlated with a significant increase in *il-10* transcription and a coordinated reduction in *il-7* and *il-15* transcription (Figure 5A).

To critically understand the role of upregulated IL-10, we next studied thymic early T-cell development in IL-10^−/−^ mice. Although the thymus was atrophied in all IL-10^−/−^ mice (regardless of tumor status), percentages of DN2 were reduced, and DN3 and DN4 subpopulations increased, in IL-10^−/−^ vs. control tumor-bearing animals (Figure 5B1). Flow-cytometric analysis supported increased Notch1 protein expression but decreased Ikaros and IRF8 protein expression by DN2a and DN2b cells isolated from tumor-bearing IL-10^−/−^ mice (Figures 5B2,B3, Supplementary Figure 1), suggesting a pivotal role of IL-10 in the reciprocal regulation of Notch1 and Ikaros signaling, leading to the arrest of T-cell maturation at the DN2 stage. *Notch1* expression was also increased and Ikaros/IRF8 expression decreased in the DN2 population isolated from tumor-bearing IL-10^−/−^ mice as compared to wild-type tumor-bearing mice (Figure 5C).

### Increased Dendritic-Cell Population and Lineage Commitment Regulated by the IL-10-Dependent Notch1/Ikaros Signaling Pathway

**Figure 6:**
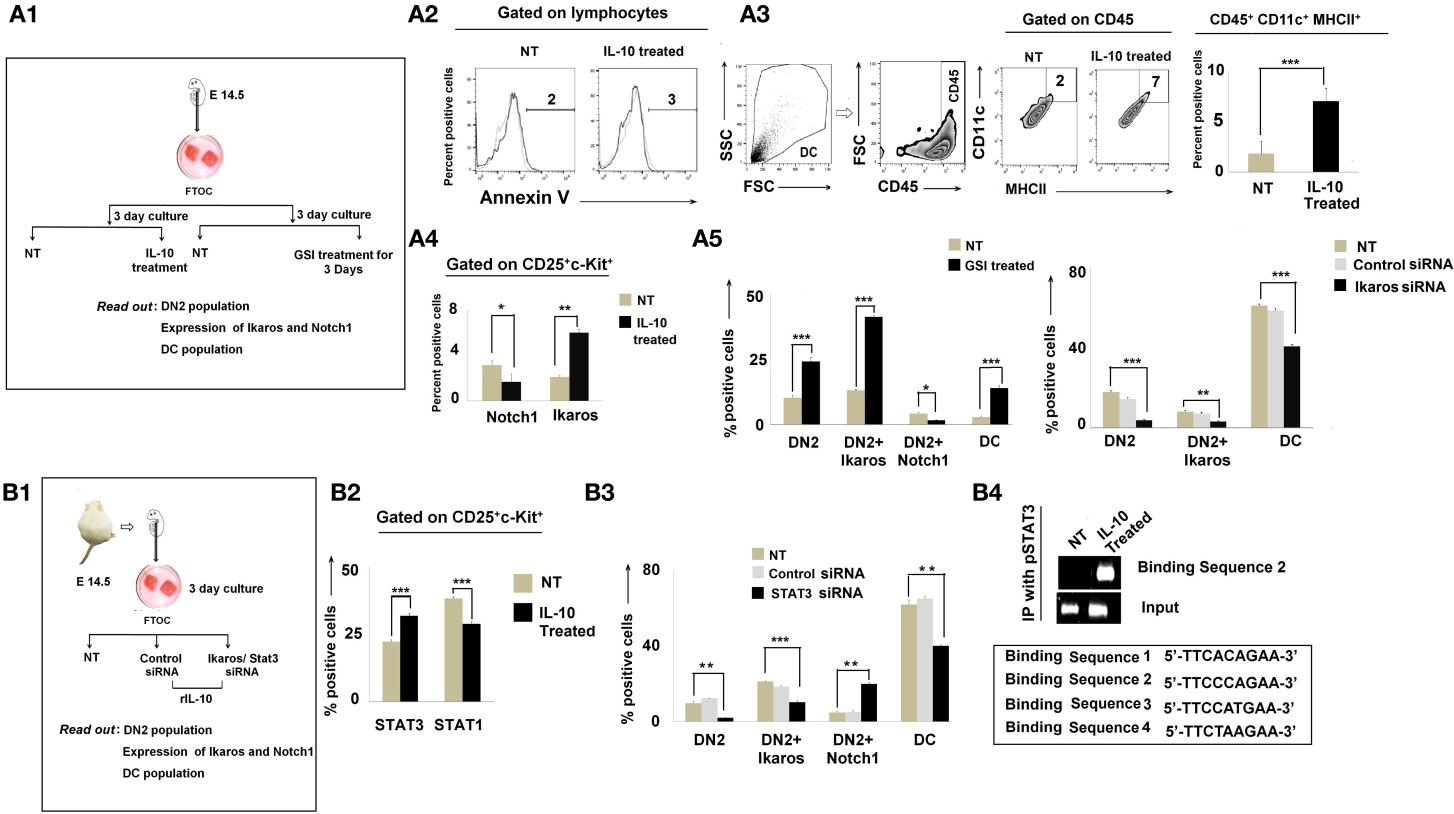
IL-10 promotes DN2-to-dendritic cell lineage differentiation by Notch1/Ikaros signaling. **(A1)** Workflow diagram of the experimental design of fetal thymic organ culture (FTOC) using the fetuses of E14.5 pregnant mice. **(A2)** Total lymphocyte populations of FTOC were analyzed by Annexin V. Histograms of Annexin V^+^ flow-cytometry results. Dotted line represents the isotype control. **(A3)** Total dendritic-cell population was checked in FTOC ± IL-10 treatment cohort. Representations of flow-cytometry results of CD11c^+^MHCII^+^ cells, gated on CD45^+^ cells, and a bar diagram showing mean ± SE of percentage positive CD45^+^CD11c^+^MHCII^+^ cells from either untreated or IL-10-treated FTOC. *n* = 3; ***p < 0.001. **(A4)** Bar diagram to represent DN2^+^Notch1^+^ and DN2^+^Ikaros^+^ cells from ± IL-10-treated FTOC, *n* = 3; *p < 0.05, **p < 0.01 **(A5)** Bar diagrammatic representations of the mean ± SE of DN2^+^, DN2^+^Ikaros^+^, and DN2^+^Notch1^+^ cells and DC population from untreated and GSI-treated cohorts in left panel along with mean ± SE of DN2^+^, DN2^+^Ikaros^+^ cells, and CD45^+^CD11c^+^MHCII^+^ DC population from untreated, control siRNA-treated, and Ikaros siRNA-treated cohorts in right panel, *n* = 3; ***p < 0.001, **p < 0.01, *p < 0.05. **(B1)** Workflow representing STAT3 and Ikaros silencing experiments with FTOC. **(B2)** Bar diagrammatic representation of mean ± SE of percentage of DN2^+^STAT3^+^ and DN2^+^STAT1^+^ cells in ± IL-10-treated cohort, *n* = 3; ***p < 0.001 **(B3)** Flow-cytometric analysis was performed; bar diagram showing mean ± SE of percentage of DN2^+^, DN2^+^Ikaros^+^, DN2^+^${\text{Notch1}}_{,}^{+}$ and CD45^+^CD11c^+^MHCII^+^ DC population from untreated, control siRNA-treated and STAT3 siRNA-treated cohorts, *n* = 3; ***p < 0.001, **p < 0.01. **(B4)** ChIP assay for pSTAT3 recruitment to the putative binding site downstream of the Notch1 promoter. Gene expression patterns of different binding sites show that upon IL-10 treatment, a specific binding site (binding sequence 2) of the downstream region of Notch1 promoter sequenced 5′ TTCCCAGAA 3′ becomes bound by pSTAT3 and Notch1 becomes downregulated.

To further understand the regulatory network formed between Notch1, Ikaros, and IL-10, we turned to analyses of *in vitro* fetal thymus organ cultures (FTOC). Fetal thymus lobes from pregnant mice (E 14.5) were cultured for 3 days (22) and treated with rIL-10 to mimic *in vivo* tumor-conditioning (Figure 6A1). In the total thymocyte population, we first checked the Annexin V expression to exclude dead cells (Figure 6A2). Corollary analyses of CD45^+^CD11c^+^MHCII^+^ cells revealed an increase in DC frequencies in IL-10-treated cultures (Figure 6A3). DN subpopulations were then analyzed based on differential expression of CD25, CD44, and c-kit. Flow-cytometric analyses revealed higher percentages of the DN2 subpopulation in IL-10-treated FTOC than in control cultures (Figure 6A4). These analyses also suggested an increased percentage of CD4^−^CD8^−^CD25^+^ c-kit^+^Ikaros^+^ cells in IL-10-treated groups, with a significant decrease in Notch1 expression by DN2 cells (Figure 6A4). Since Notch1 expression was reduced in IL-10-conditioned early arrest of T cells in FTOC, we next evaluated the impact of adding gamma secretase inhibitor-953 (Cpd11) to FTOC to block Notch1 downstream signaling (Figure 6A4) (40). Consistent with our evolving operational paradigm, inhibition of Notch1 signaling resulted in arrest in DN2 populations while increasing Ikaros expression and promoting the accumulation of CD45^+^CD11c^+^MHCII^+^ DCs in FTOC (Figure 6A5). Next, to validate the association between upregulated expression of Ikaros and termination in T-cell lineage commitment and its differentiation to DC, we performed knockdown of *ikaros* by ikaros-specific siRNA. A significant absence of ikaros (by 50%) results in a decrease in the DC population with a simultaneous release in the arrest of the DN2 population, which normalizes lineage commitment (Figure 6A5, Supplementary Figure 2).

Since STAT3 is essential in IL-10 signaling, we further decided to assess the impact of silencing STAT3 using specific siRNA in FTOC (Figures 6B1,B2). Silencing of STAT3 was determined to significantly ameliorate the regulatory effects of IL-10, leading to the rescue of (normal) DN2 → DN3 transition in concert with normalized Notch1 and Ikaros expression (Figure 6B3).

As IL-10 failed to induce DN2 → DN3 arrest in the absence of STAT3 (in concert with upregulated Notch1 expression), we next investigated the nucleotide sequence of the mouse *notch1* gene for the presence of putative STAT3 binding sites. In the nucleus, activated and tyrosine-phosphorylated STAT3 binds to the DNA-response elements (i.e., interferon-γ-activated sequence; GAS) found in the promoter regions of target genes (41). GAS is a nine-base-pair palindrome, having the consensus sequence TTCCGGGAA. Interestingly, we found four sites within introns just after the promoter region having the sequence 5′-TTCACAGAA-3′ from 991 to 999 bp (first site), 5′-TTCCCAGAA-3′ from 3,179 to 3,187 bp (second site), 5′-TTCCATGAA-3′ from 5,773 to 5,781 bp (third site), and 5′- TTCTAAGAA-3′ from 10,583 to 10,591 bp (fourth site) that were highly similar to the consensus GAS sequence (Figure 6B4). Accordingly, we performed ChIP assays to examine the binding of pSTAT3 to the *notch1* gene. Unstimulated control DN2 T cells show virtually no binding of pSTAT3 to the *notch1* gene at any site, while IL-10 pretreatment significantly augmented binding of pSTAT3 to the *notch1* gene at the second site, i.e., from 3,179 to 3,187 bp (Figure 6B4). These data suggest that IL-10-conditioning promotes direct binding of pSTAT3 to the GAS motif of the *notch1* gene, leading to reduced *notch1* gene expression and the differentiative arrest of DN2 T cells.

### IL-10-Treated Stromal Cells Reprogram IL-10R^High^ DN2 Cells Toward DC Differentiation in the Tumor-Bearing Host

**Figure 7:**
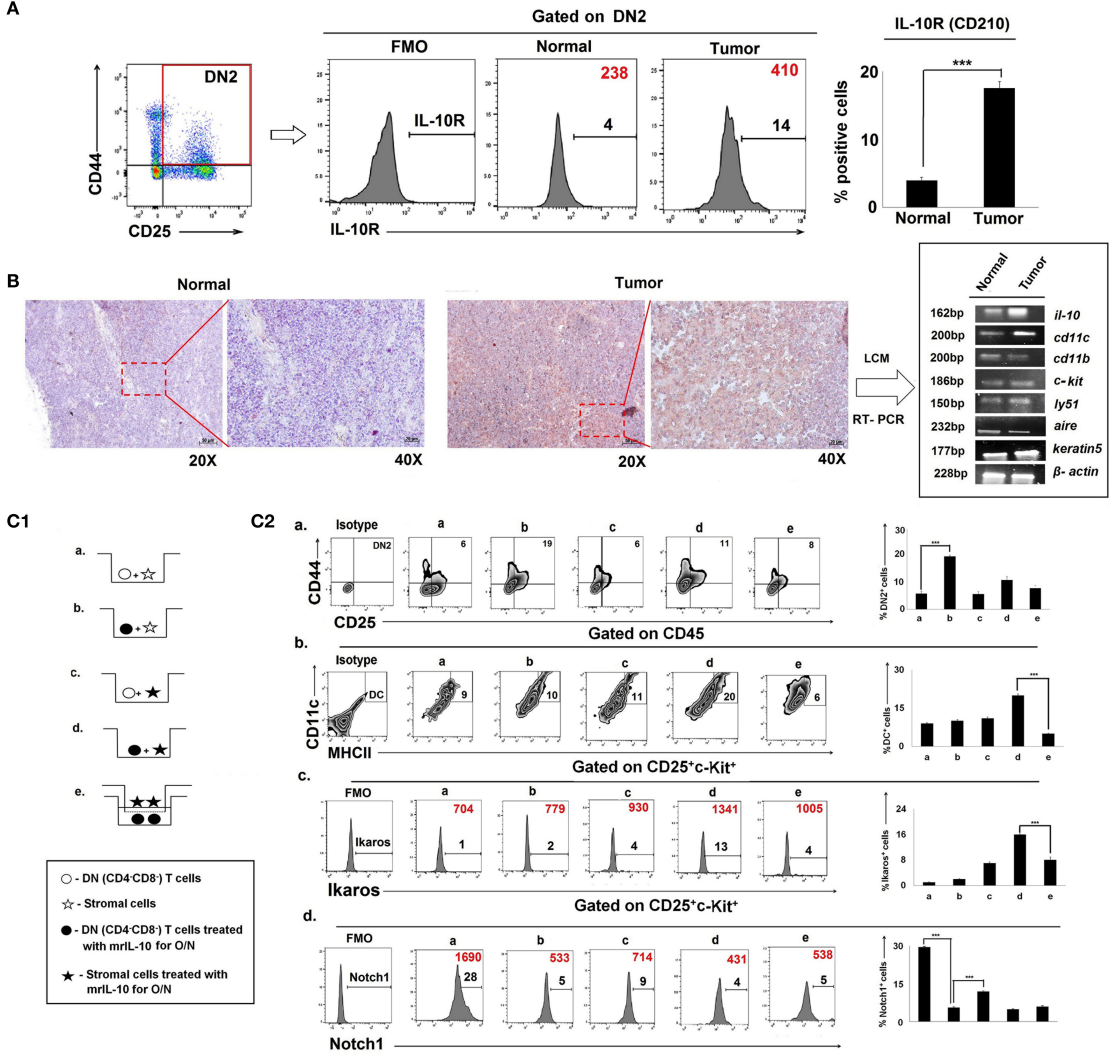
Physical interaction between IL-10R^high^ T cells and stromal cells is required in tumor-induced early arrest and switching of DN2 pro-T cells toward DCs: **(A)** IL-10 receptors in thymic DN2 T cells are represented by flow-cytometric histograms (positive percentages are written in black, and MFI values are written in red). Bar diagram represents mean of positive percentages ± SE; *n* = 3, ***p < 0.001. **(B)** IL-10-rich zone in thymi of normal and tumor hosts was identified immunohistochemically. IL-10-rich regions, i.e., cortico-medullary region of thymus, are shown in 20 × and 40 × magnification, and these zones were isolated using a laser capture microscope to analyze the gene expression using mRNA by RT-PCR, keeping β-actin as a loading control. Genes include *il-10, cd11c, cd11b, c-kit, ly51, aire*, and *keratin5* in samples from normal and tumor cohorts (*n* = 3 in each case). **(C1)** Pictorial diagram of experimental set up of T-cell and stromal-cell interaction. DN T cells were isolated by BD Imag, and stromal cells were isolated by 2-deoxyguanosine treatment in FTOC culture for 3 days. DN-T cells and stromal cells were then co-cultured in transwell with or without trans-membrane and IL-10 treatment. Different conditions are: (a) DN T cells were co-cultured with stromal cells, (b) DN T cells were pre-treated with mrIL-10 overnight and co-cultured with stromal cells, (c) stromal cells were pre-treated with mrIL-10 overnight and co-cultured with DN T cells, (d) stromal cells and DN T cells were pre-treated with mrIL-10 overnight and then co-cultured, (e) stromal cells and DN T cells pre-treated with mrIL-10 overnight, and then DN T cells co-cultured with stromal cells were separated with 0.8-μm trans-membrane in trans-well. **(C2)** Flow cytometric representations of thymic cells for (a) CD25^+^CD44^+^, (b) CD45^+^CD11c^+^MHCII^+^, (c) CD25^+^c-Kit^+^Ikaros^+^ (d) CD25^+^c-Kit^+^Notch1^+^ from (a–e) culture conditions as mentioned. In histograms, MFI values are represented in red, and positive percentages of cells are presented in black. Bar diagrams in each figure show the mean ± SE of 3 individual observations; ***p < 0.001.

Since IL-10 controlled early arrest of DN2 T cells, we profiled IL-10 receptor expression on DN1-DN4 cells. Flow-cytometric analysis revealed an increase in the expression of IL-10R (CD210) in Lin-thy1.2^+^CD25^+^CD44^+^ DN2 T-cells isolated from the thymus of tumor-bearing vs. control, tumor-free mice (Figure 7A). An immunohistochemical analysis of the IL-10 protein expression pattern in thymus suggested focused expression in cortico-medullary junctions (Figure 7B), where accumulation of early T cells is commonly observed. Laser capture micro-dissection of the IL-10^high^ region followed by mRNA analysis suggested keratin5^+^ thymic cortical epithelial cells as a major source of IL-10, along with CD11c^+^ thymic DCs (Figure 7B).

In order to examine whether thymic epithelial cell (TEC)-secreted IL-10 was sufficient to induce both blockade and lineage-switching in early T-cell developmental programming, we established *in vitro* co-culture trans-well assays using purified populations of DN2 cells and stromal cells in the presence and absence of rmIL-10 (Figure 7C1, Supplementary Figure 3). We observed that while rmIL-10 alone was moderately effective in promoting the arrest of DN2 cells (Figure 7C2), it failed to promote the T-DC differentiative switching. Maximum DN2 arrest and coordinate T-DC differentiation were observed only when IL-10 was used to pretreat stromal cells that were then co-cultured with IL-10-pretreated DN T cells. Physical separation of DN T cells from stromal cells had no effect on T-cell maturation or in early T-cell arrest (Figure 7C2). Therefore, physical interaction between IL-10-conditioned stromal cells and DN T cells is required for DN2 → DN3 arrest and the redirection of DN2 → DC differentiation programming.

## Discussion

**Figure 8:**
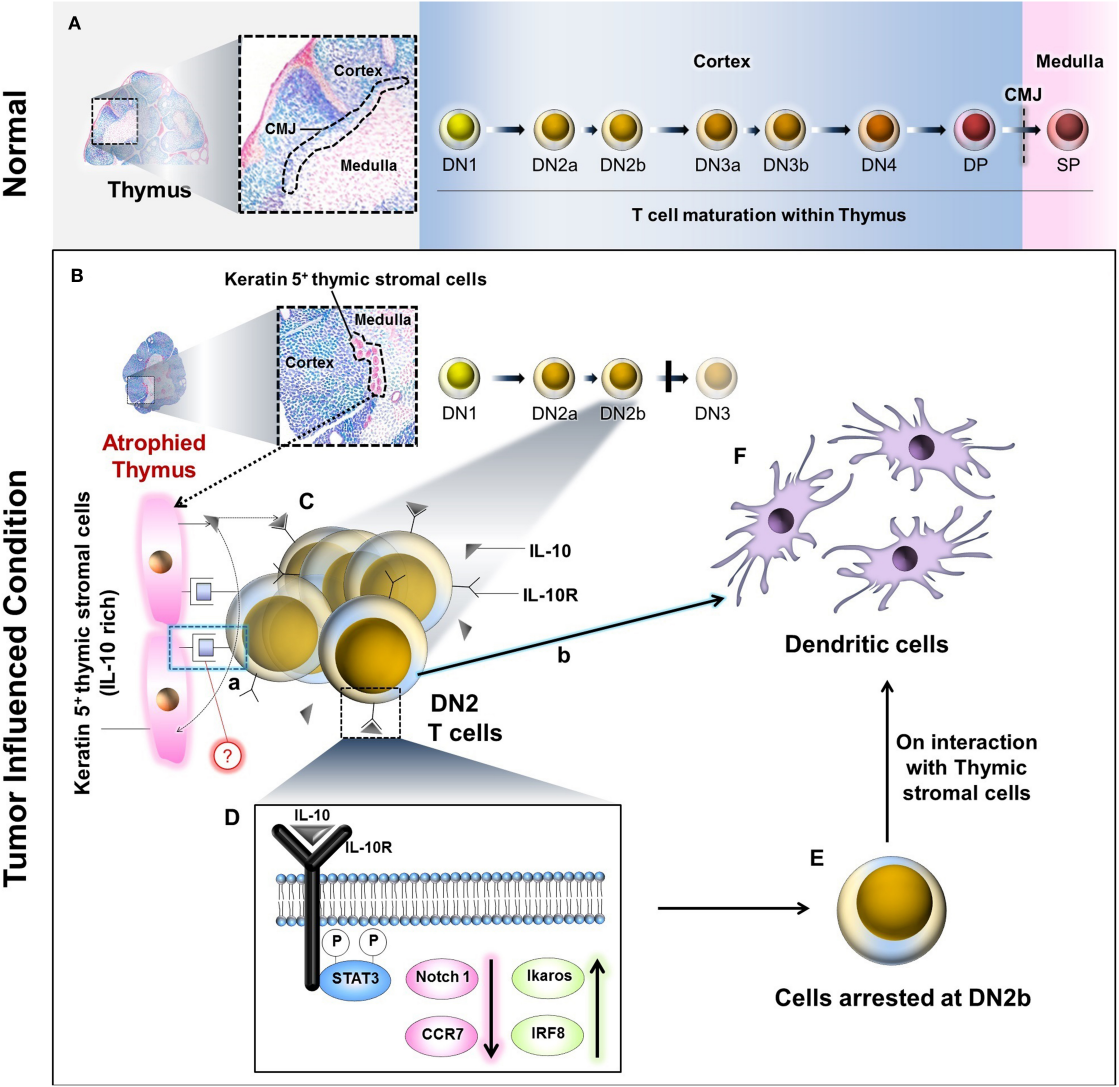
Schematic diagram and probable mechanism of arrest of DN2 pro-T cell-to-DN3 transition and promotion of its conversion to thymic dendritic cells by reciprocal regulation of Ikaros/Notch1 signaling: **(A)** During maturation, the T lymphocytes progress through the DN1, DN2a, DN2b, DN3a, DN3b, and DN4 to DP stages within the thymic cortex. These DP cells pass the cortico-medullary junction (CMJ) to enter the medulla as they further mature to SP (DN, Double negative; DP, Double positive; SP, Single positive). **(B)** Under the influence of tumor, thymus becomes atrophied, and Keratin-5 expressing IL-10-rich stromal cells tend to accumulate at the CMJ. **(C)** IL-10 molecules secreted from these cells interact with DN2b T cells via IL-10 receptors (IL-10R). **(D)** However, following IL-10–IL-10R interaction(s), STAT3-mediated intracellular signaling events occur within the DN2 T cells, which ultimately bring down Notch1 expression. As a consequence, the T cells are arrested at the DN2b stage. **(E)** Other than Notch1, downregulation of CCR7 and upregulation of Ikaros and IRF8 has also been observed due to these signaling events. A few of these arrested DN2b T cells somehow interact physically with the Keratin 5^+^ thymic stromal cells at the CMJ (C.a**)** and differentiate into dendritic cells (C.b, **F**) instead of staying at DN2b or progressing to DN3. The precise mode of the physical interaction(s) between the DN2b T cells and the Keratin-5^+^ stromal cells (C.a) is yet to be elucidated.

Progressively growing tumors in both human and mice are associated with host thymic atrophy, leading to alterations in T-cell proliferation, apoptosis, and/or differentiation programming, yielding paralysis in adaptive anti-tumor immunity (8, 42). Although the thymus undergoes age-related senescence after puberty, recent data suggest that extraneous stimuli, including inflammation, can reinvigorate thymopoiesis (6). One caveat in such thymic reactivation is that resultant T cells may be predominantly regulatory (Treg) vs. effector (T_eff_) in nature, a detriment to effective host protection against cancer. In the present study, our major findings include the following. (i) Tumor progression is associated with an early arrest in transition between the DN2 to DN3 stages of T-cell maturation in the thymus. (ii) Tumor-induced IL-10 interferes with the interaction between thymic stromal cells and IL-10R^high^ DN2 T cells to arrest their development. (iii) IL-10 counter-regulates Notch1 and Ikaros/IRF8 signaling while suppressing the expression of Notch1 target gene *ccr7* on DN2 T cells. (iv) This process instead shunts DN2b T-cell commitment toward DC differentiation (Figure 8).

T-cell maturation in the thymus is initiated from Lin^−^CD4^−^CD8^−^CD25^−^CD44^+^c-Kit^+^DN1 precursor cells, which subsequently pass through an ordered series of developmental stages, namely DN1-DN2-DN3-DN4-DP-SP, to maintain the supply of mature T cells circulating in the periphery of the host. Thymic T-cell output is drastically reduced in the vast majority of malignant diseases (43). In line with this observation, we noted an accumulation of CD4^−^CD8^−^(DN) thymocytes in tumor-bearing host in total thymocytes and in the lineage negative and thy1.2 positive population (Supplementary Figure 5). Consistent with previous reports (27), we observed severe thymic atrophy across a range of murine tumor models. Indeed, Adkins et al. (44) previously reported thymic T-cell arrest at an early stage (CD25^+^CD44^+^ DN2) in breast carcinoma-bearing mice. Here, we refined such analyses by the inclusion of an additional marker, c-Kit, providing evidence for prominent arrest occurring between the DN2b and DN3 stages of pro-T-cell development, resulting in the accumulation of CD25^+^CD44^+^c-Kit^low^ DN2b cells within the cortico-medullary junction and depletion of more mature DN3 and DN4 T cells. During tumor progression, the thymic T-cell differentiation process appears to be negatively impacted by the silencing of Notch1 signaling (45) and corollary expression of Notch1-downstream target *ccr7* (19, 46), along with its two ligands CCL19 and CCL21 within thymus. In this light, previous reports have suggested an indispensable role of CCR7 in facilitating the migration of the CD25^+^CD44^+^ DN2 population to the outer thymic cortex (47), with CCR7 deficiency restricting these cells within the cortico-medullary junction (CMJ) and hampering their development beyond the DN2 stage (28, 48). In FTOC cultures, under conditions permissive for Notch1 signaling (i.e., with TNFα stimulation), DN2-to-DN3 transition was promoted. However, when Notch1 signaling was blocked by a gamma-secretase inhibitor, early T-cell differentiation was prevented.

In extended studies, we determined that another important contributor to tumor-induced T-cell developmental arrest is IL-10, whose expression was upregulated in the thymus, particularly that of the tumor-bearing host, particularly at the thymic CMJ where DN2 T cells tend to accumulate. Although previous reports suggest that tumor-associated IL-10 activity disrupts normal T-cell maturation (49), even in some SCID patients, overexpression of IL-10 appears to specifically interrupt T-cell maturation at an early stage (50). However, these studies failed to explain how tumor initiation and/or progression promoted intra-thymic IL-10 activity. Clearly, one cannot ignore the additional contributions of one or several tumor-induced systemic factor(s) including hormones (secreted from tumor-involved organs), neurotransmitters, galectins, and PGE2 in intra-thymic cytokine (including IL-10)-production by TEC. In our model, keratin5^+^ medullary TEC seemed to be primarily responsible for IL-10 production in the tumor-bearing host, followed by CD11c^+^ DCs. Not surprisingly, IL-10R expression was determined to be higher in the DN2 T-cell subset, likely making IL-10R^high^ DN2 T cells more susceptible to IL-10-mediated lineage arrest. IL-10 was also likely responsible for suppressing the Notch1 expression in DN2a T cells, since *notch 1* expression in DN2 T cells and transition from DN2 to DN3 was normalized in tumor-bearing IL-10^−/−^ mice. To support the notion that IL-10-mediated arrest at the DN2-to-DN3 transition involves STAT3, we showed that knockdown of STAT3 (using STAT3 specific siRNA) ameliorates IL-10-induced arrest at the DN2 stages in concert with normalization in *notch1* expression. In contrast to our observations, Garner et al. (51), reported that constitutively activated STAT3 along with activated NF-κB promotes Notch expression in glioblastoma cancer stem cells. In our hands, however, we failed to see any activation in NFκB-associated molecules, which may simply be related to the different cell systems being evaluated in each case. Therefore, to better understand the regulatory effects of IL-10 on *notch1* more precisely, we designed four primers based on the putative *p*STAT3 binding site downstream of *notch1* promoter. ChIP assays performed on sorted DN2 T cells supported the direct binding of pSTAT3 at a 5′-XXX-TTCCAGAA-XX-3′ site downstream of the *notch1* promoter (among the four putative stat3 binding regions) of *notch1* gene after IL-10 stimulation. Interestingly, unlike Notch1, Ikaros and IRF8 expressions were found to be significantly elevated in the “stunted” DN2-T cells isolated from the thymi of tumor-bearing mice. Moreover, FTOC experiments suggested that the reciprocal regulation of Notch1 and Ikaros/IRF8 is IL-10 dependent. In the normal thymus, Ikaros expression was restricted primarily to DN3 and DN4 T cells, where it is believed to play a critical role as a checkpoint regulator during DN3 to DN4 transition and again during subsequent DN4 to DP transition. Furthermore, loss of Ikaros in the face of intact Notch1 signaling allows for T-cell maturation programming to occur between the DN-DP and DP-SP transition stages without appropriate pre-TCR and TCR signaling (52). In contrast, in a tumor-conditioning model, enhanced *Ikaros* in the absence of *notch1* along with *tcf1* and *bcl11b* (18, 53, 54), leads to aborted T-cell maturation beyond DN2a. Accordingly, knockdown of Ikaros promotes pro-T-cell maturation beyond the DN2 stage and a reduction of the DC population, like in a tumor-free host.

Strikingly, we observed a significant rise in lymphoid DC frequencies in thymus of tumor-bearing mice without discernable alterations in other immune cell subpopulations. Thymic pro-T cells, through many generations, retain the differentiative potential for alternate cell lineages, such as monocytes or DC (39), Such latent myeloid differentiative potential in committed T cells is also promoted by ectopic anti-inflammatory cytokine production (55) and by the dysregulation of transcription factors such as Notch1 (39, 56, 57). Therefore, loss of Notch1 induced by IL-10, yields a conditional state conducive for DN2 T cell → lymphoid DC differentiation [i.e., coordinated expression of Pu.1 (58), Cebpalpha (59), Ikaros, and IRF8 along with CD3ε] within the thymus of the cancer-bearing host. This inter-lineage conversion of DN2 T cells is consistent with a report from Feyerabend et al.; however, unlike their observation, notch1 reduction in a tumor-induced model only resulted in the generation of lymphoid DCs but not B cells, and the involvement of Ikaros and IRF8 was not demonstrated. Notably, the role of Ikaros and IRF8 in promoting DC commitment in the extra-thymic environment has been reported previously (60–62). DC lineage potential is clearly present in ETPs and DN2a cells (63–65), and when we specifically checked the DN2a and DN2b populations, we found that *ikaros* and *pu.1* expression became elevated in the DN2a stage, which may serve as a preparatory phase for initiation of arrest in T cell-lineage commitment and switching to DC, which drives DN2b to DC commitment in tumor host. Moreover, we also checked the contribution of homing of circulating DC via CD49d; however, neutralization of CD49d ruled out such a possibility.

Additionally, in this trans-differentiation process, direct cell-to-cell interactions between DN2 thymocytes and IL-10-pretreated thymic epithelial cells are required (66), as IL-10 treatment of DN-T cells only partially restricts DN T-cell maturation at the DN2 stage, and it failed in DN2 → DC differentiation. This observation again strongly supports the influence of lymphoid-stromal cell interactions as a major determinant for lineage commitment (67); which stromal cells are responsible for this effect will be determined in future studies.

In conclusion, we have identified a novel mechanism through which (tumor-induced) IL-10 paralyzes host anti-tumor immunity by blocking thymic DN2a cells along a T-cell fate pathway and altering the T-cell fate pathway by promoting the commitment of pro-T (DN2) toward DC lineage. This mechanism of lineage re-registry is unique to the cancer setting and distinct from age-induced thymic involution. Finally, the role(s) of thymic DC evolved from DN2 T-cell progenitors in the evolving anti-tumor T-cell repertoire and tumor progression remain unknown, providing a major area of focus for future studies designed to advance our understanding of tumor-associated immune deviation and the development of target therapeutics for improved treatment outcomes in the cancer setting.

## Data Availability Statement

The data and materials related to the findings of this study are mentioned in the article, figures, and Supplementary Material. Raw data are available from the corresponding authors on reasonable request.

## Ethics Statement

The animal study was reviewed and approved by Institutional Animal Care and Ethics Committee of CNCI, Kolkata, India. Ethics Committee Approval No. IAEC-1774/RB-4/2015/6 and IAEC-1774/RB-19/2017/15.

## Author Contributions

IG, ABo, and RB designed the study, analyzed the data, and wrote the manuscript. IG, ABh, DS, AP, PN, AS, and SD performed the research. SG, BS, and AN provide resources. SM, BS, and WS prepared the manuscript. ABo and RB supervised the project. ABo and RB acquired the funding. All authors approved the manuscript.

## Conflict of Interest

The authors declare that the research was conducted in the absence of any commercial or financial relationships that could be construed as a potential conflict of interest.

## Supplementary Material

Supplementary Figure 1 Overall gating strategy of CD45, CD4, CD8, CD25, CD44, c-Kit, Notch1, Ikaros, and IRF8 stained thymic T cells from non-tumor (wild-type and IL-10^−/−^) and tumor (wild-type and IL-10^−/−^) hosts. Dot plot representations for **(A)** lymphocytes, **(B)** doublet discrimination, **(C)** CD45 gating, with CD45^+^ population selected on lymphocytes, **(D)** CD4/CD8 gating, **(E)** DN (CD4^−^CD8^−^) population was selected for DN1, DN2, DN3, and DN4 analysis based on CD25 CD44-staining. **(F)** DN2 population was sub-gated into DN2a and DN2b based on c-Kit, CD25 expression. **(G)** Histograms represent FMO of Ikaros^+^, IRF8^+^, and Notch1^+^ cells within DN2a and DN2b cell populations, respectively, *n* = 3.

Supplementary Figure 2 **(A)** Flow-cytometric analysis of Ikaros was performed in untreated and Ikaros siRNA-treated cohorts. Ikaros was analyzed on DN2 (CD25^+^CD44^+^) positive cells. Histograms represent percentage of positive cells of FMO, untreated, and Ikaros siRNA-treated cohorts, respectively, on DN2^+^ cells.

Supplementary Figure 3 **(A)** Workflow diagram on experimental design of 2-deoxy guanosine treatment on fetal thymic organ culture (FTOC) using E14.5 fetus from IL-10^−/−^ pregnant mice and co-cultured with wild-type fetal thymocytes. **(B)** Bar diagram shows the percentage of DN2, DC, DN2^+^Ikaros and DN2^+^Notch1 positive cells of untreated and IL-10-treated cohorts; *n* = 3, ^***^*p* < 0.001.

Supplementary Figure 4 **(A)** Flow-cytometric dot plot representations of thymic cells with CD4 and CD8 staining from Days 0, 1, 3–7 of FTOC culture. **(B)** Bar diagrams represent percentages of DN, DP, CD4SP, and CD8SP cells in total FTOC population at Days 4–6; *n* = 4, *p* < 0.001.

Supplementary Figure 5 **(A)** Bar diagram represents the pre- and post-sorting percentages of DN, DP, CD4SP, and CD8SP thymocyte positive cells. **(B)** Bar diagram represents the pre- and post-sorting absolute cell numbers of DN, DP, CD4SP, and CD8SP thymocytes. In the case of pre-sorting, absolute numbers of thymocytes were counted from total cell population, and in the case of post-lin^−^Thy1.2^+^-sorting, absolute numbers were calculated from total sorted population for DN, DP, CD4SP, and CD8SP thymocytes; *n* = 4, *p* < 0.001.

## Abbreviations

DN: Double negative
DP: Double positive
SP: Single positive
DC: Dendritic cell
S180: Sarcoma 180
FTOC: Fetal thymic organ culture
IRF8: Interferon Regulatory Factor 8
Lin^−^: Lineage negative
CMJ: Cortico medullary junction
dGuo-2′: deoxyguanosine
BrdU: 5′-Bromo-2′-deoxyuridine
TEC: Thymic epithelial cell.
