# Supplementary figures and images for: Tumor Arrests DN2 to DN3 Pro T Cell Transition and Promotes Its Conversion to Thymic Dendritic Cells by Reciprocally Regulating Notch1 and Ikaros Signaling
Source: Front Immunol. 2020 Jun 5;11:898. doi: 10.3389/fimmu.2020.00898 (PMC7292239; doi:10.3389/fimmu.2020.00898)

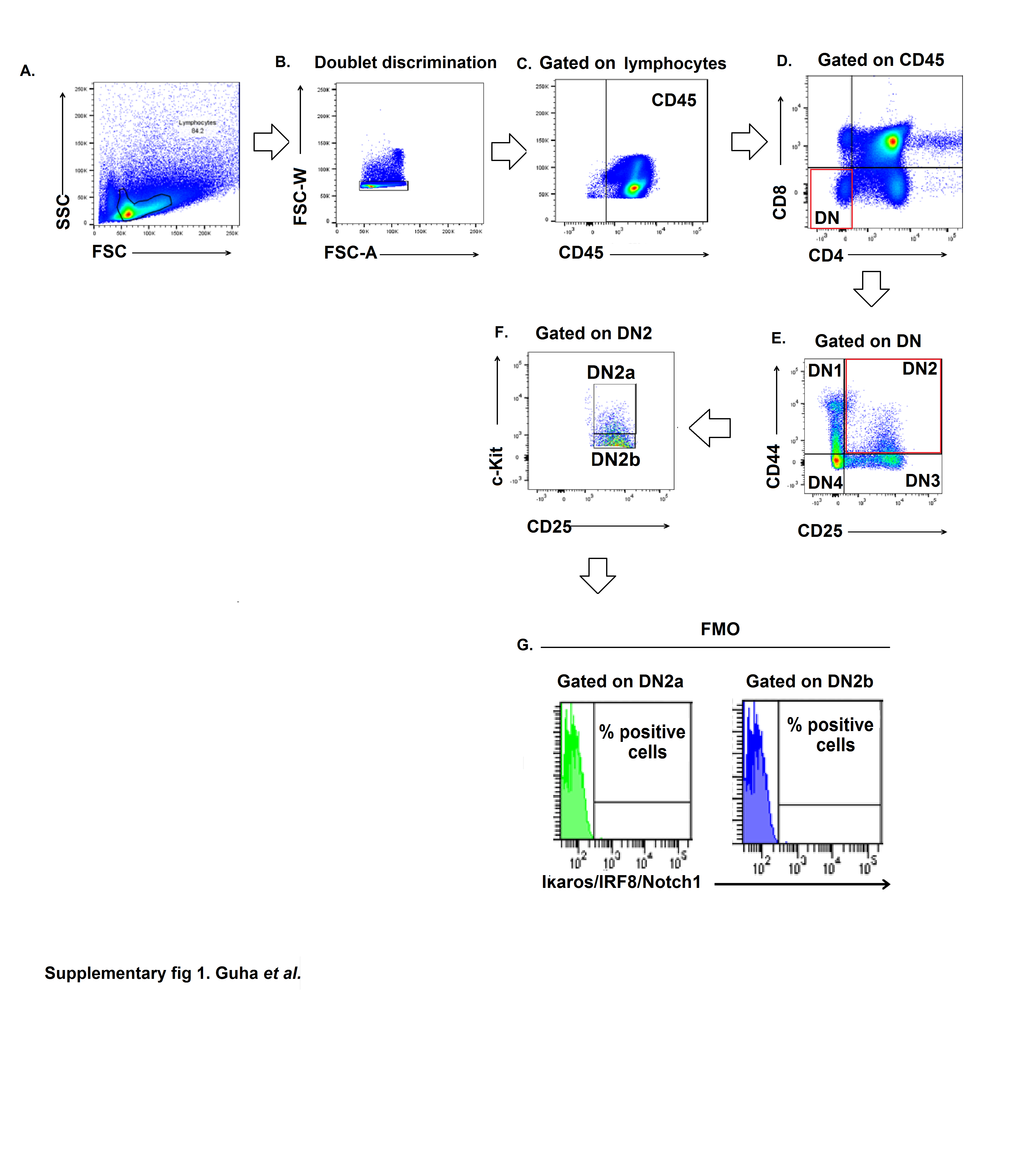

Supplement: Supplementary Figure 1 — Overall gating strategy of CD45, CD4, CD8, CD25, CD44, c-Kit, Notch1, Ikaros, and IRF8 stained thymic T cells from non-tumor (wild-type and IL-10−/−) and tumor (wild-type and IL-10−/−) hosts. Dot plot representations for (A) lymphocytes, (B) doublet discrimination, (C) CD45 gating, with CD45+ population selected on lymphocytes, (D) CD4/CD8 gating, (E) DN (CD4−CD8−) population was selected for DN1, DN2, DN3, and DN4 analysis based on CD25 CD44-staining. (F) DN2 population was sub-gated into DN2a and DN2b based on c-Kit, CD25 expression. (G) Histograms represent FMO of Ikaros+, IRF8+, and Notch1+ cells within DN2a and DN2b cell populations, respectively, n = 3. [file Image_1.tif]

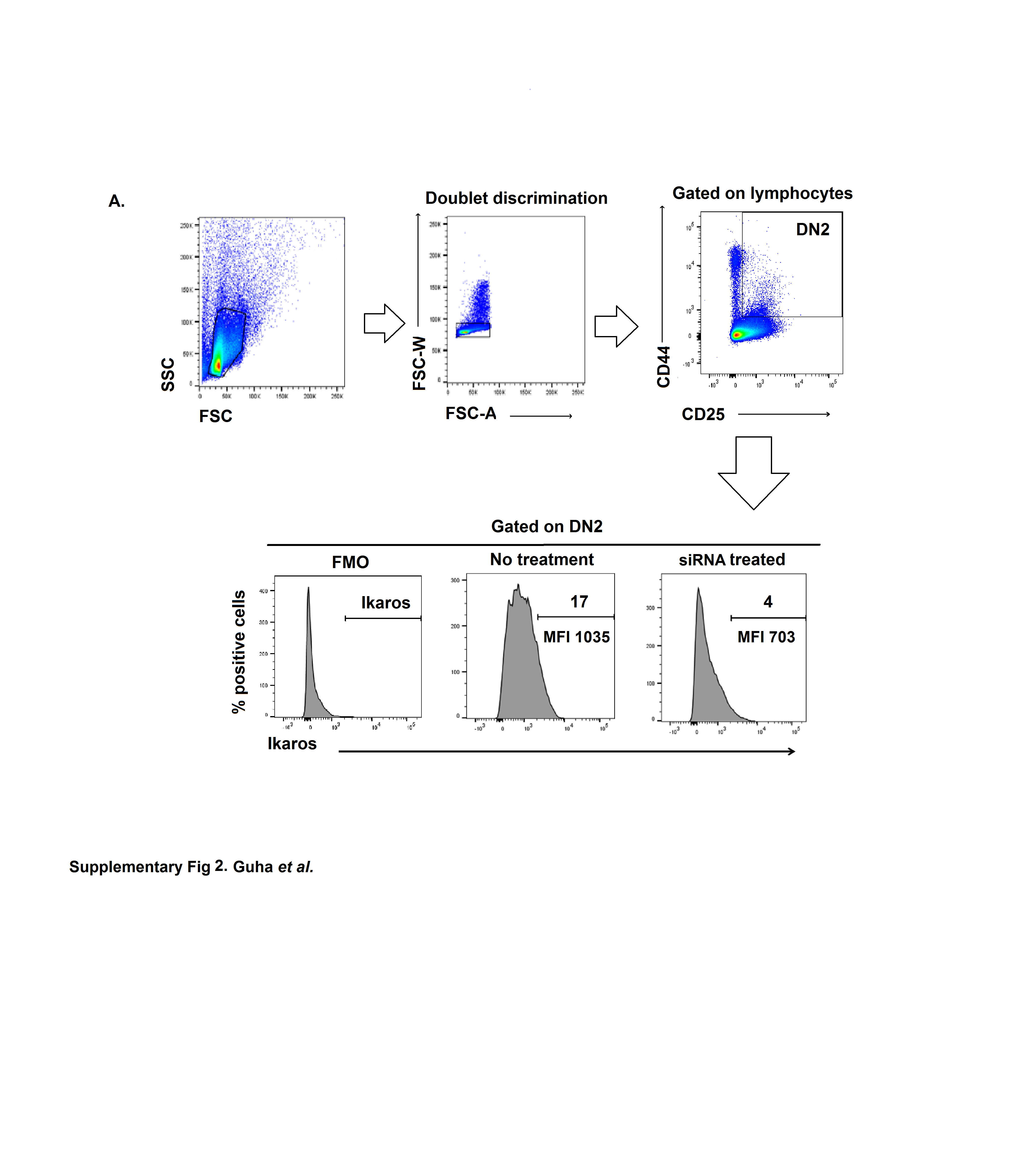

Supplement: Supplementary Figure 2 — (A) Flow-cytometric analysis of Ikaros was performed in untreated and Ikaros siRNA-treated cohorts. Ikaros was analyzed on DN2 (CD25+CD44+) positive cells. Histograms represent percentage of positive cells of FMO, untreated, and Ikaros siRNA-treated cohorts, respectively, on DN2+ cells. [file Image_2.tif]

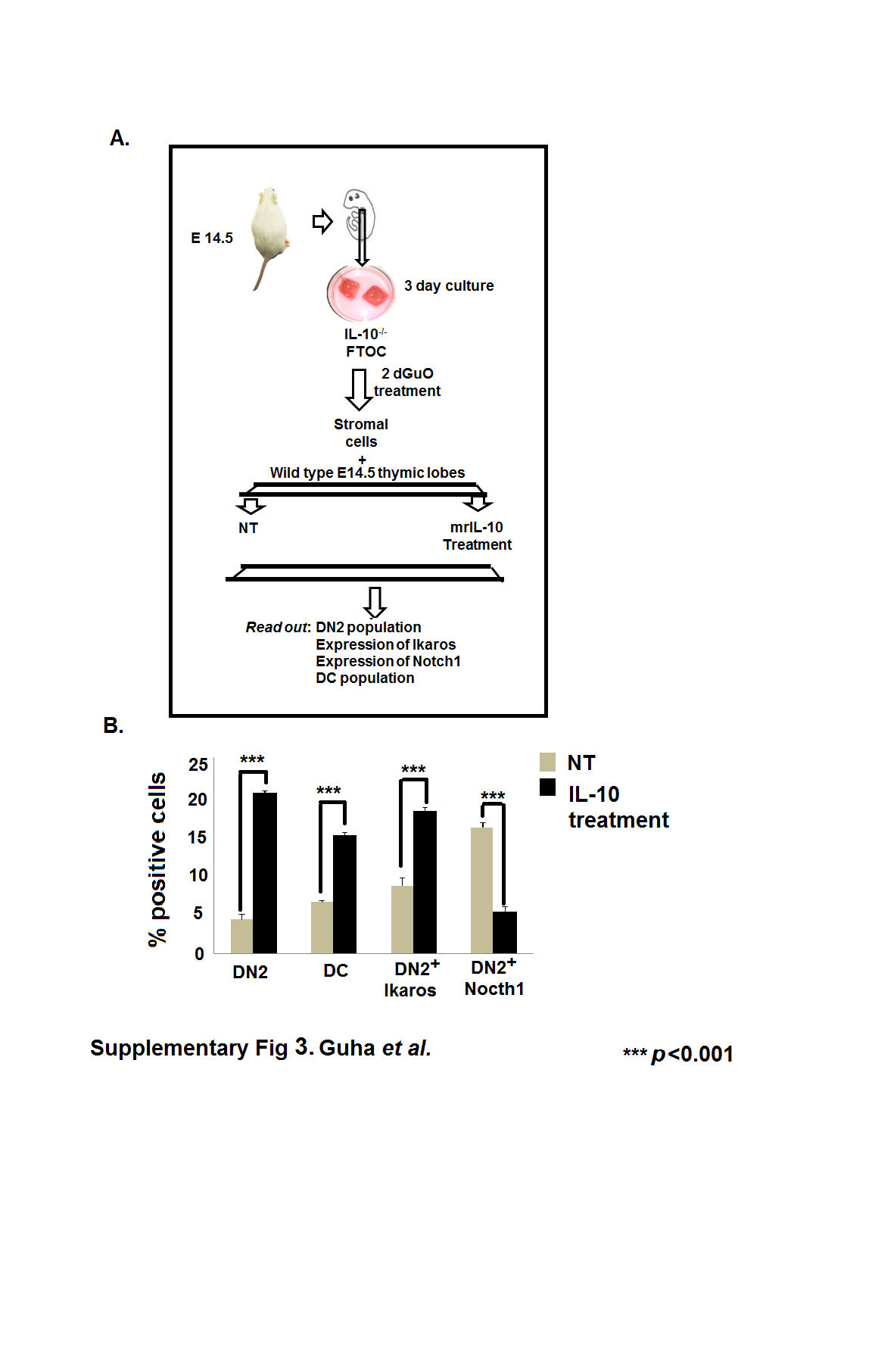

Supplement: Supplementary Figure 3 — (A) Workflow diagram on experimental design of 2-deoxy guanosine treatment on fetal thymic organ culture (FTOC) using E14.5 fetus from IL-10−/− pregnant mice and co-cultured with wild-type fetal thymocytes. (B) Bar diagram shows the percentage of DN2, DC, DN2+Ikaros and DN2+Notch1 positive cells of untreated and IL-10-treated cohorts; n = 3, ***p < 0.001. [file Image_3.tif]

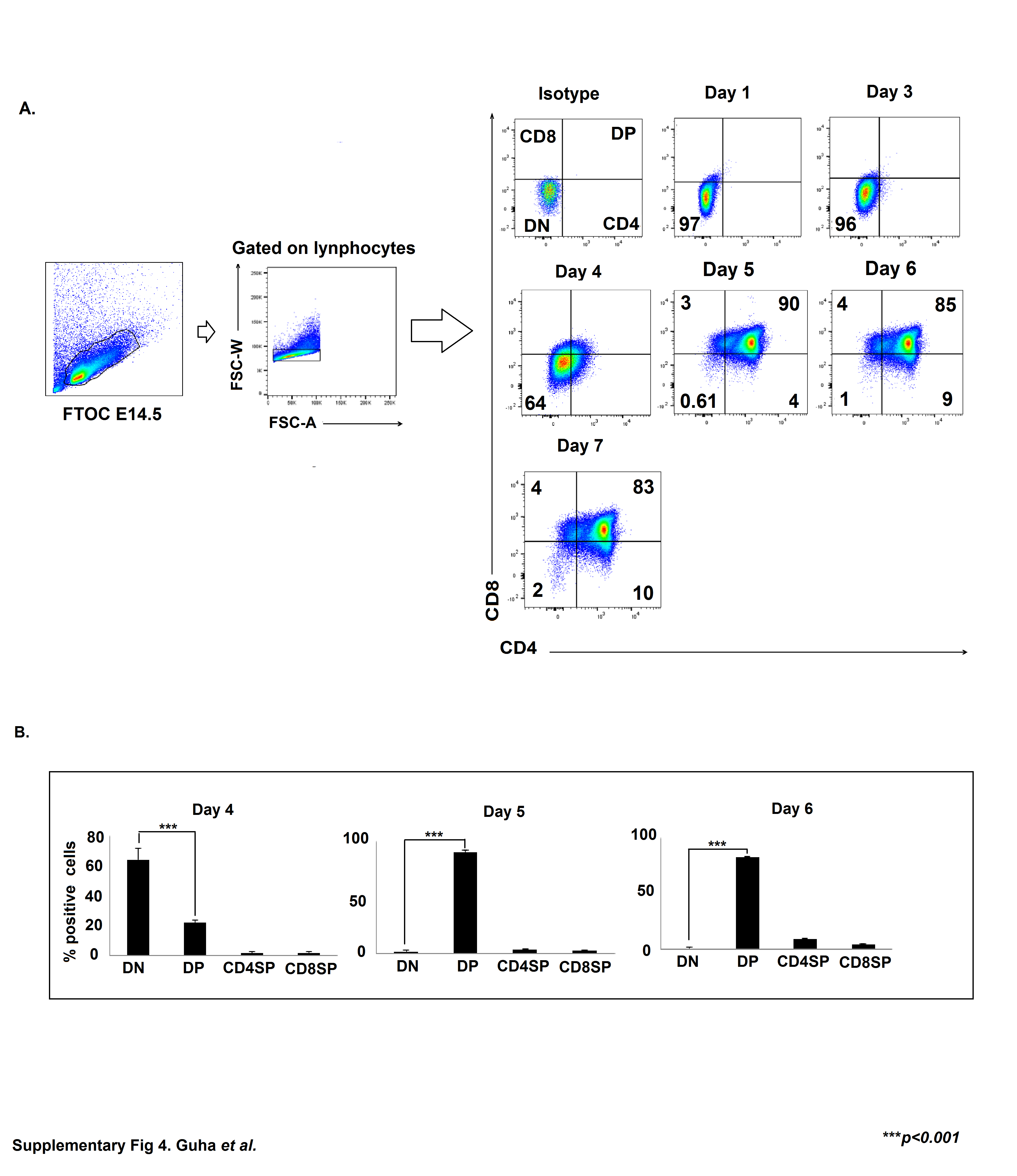

Supplement: Supplementary Figure 4 — (A) Flow-cytometric dot plot representations of thymic cells with CD4 and CD8 staining from Days 0, 1, 3–7 of FTOC culture. (B) Bar diagrams represent percentages of DN, DP, CD4SP, and CD8SP cells in total FTOC population at Days 4–6; n = 4, p < 0.001. [file Image_4.tif]

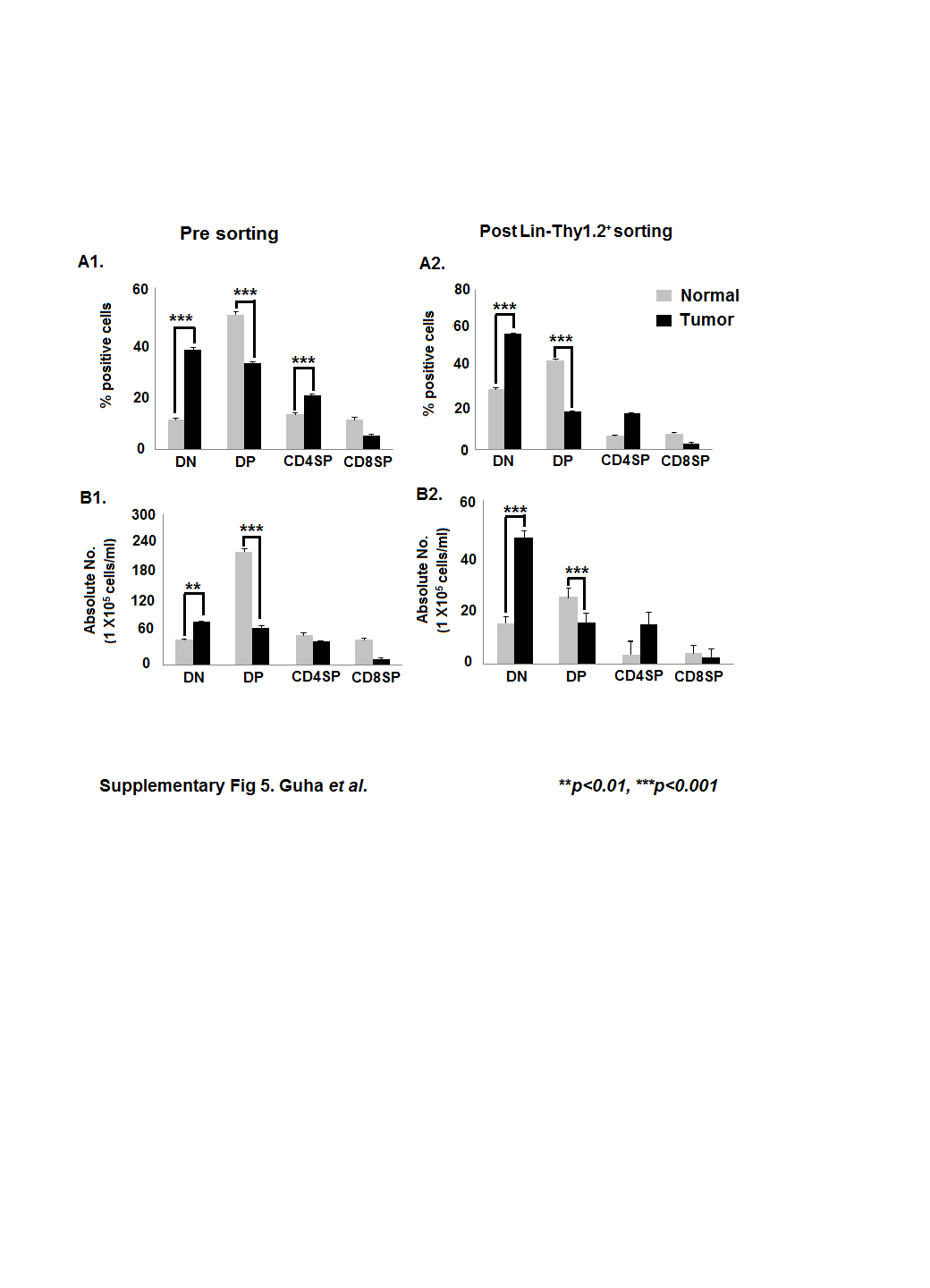

Supplement: Supplementary Figure 5 — (A) Bar diagram represents the pre- and post-sorting percentages of DN, DP, CD4SP, and CD8SP thymocyte positive cells. (B) Bar diagram represents the pre- and post-sorting absolute cell numbers of DN, DP, CD4SP, and CD8SP thymocytes. In the case of pre-sorting, absolute numbers of thymocytes were counted from total cell population, and in the case of post-lin−Thy1.2+-sorting, absolute numbers were calculated from total sorted population for DN, DP, CD4SP, and CD8SP thymocytes; n = 4, p < 0.001. [file Image_5.tif]
